# Epigenetic Analysis of KSHV Latent and Lytic Genomes

**DOI:** 10.1371/journal.ppat.1001013

**Published:** 2010-07-22

**Authors:** Zsolt Toth, Dennis T. Maglinte, Sun Hwa Lee, Hye-Ra Lee, Lai-Yee Wong, Kevin F. Brulois, Stacy Lee, Jonathan D. Buckley, Peter W. Laird, Victor E. Marquez, Jae U. Jung

**Affiliations:** 1 Department of Molecular Microbiology and Immunology, Keck School of Medicine, University of Southern California, Los Angeles, California, United States of America; 2 USC Epigenome Center, Keck School of Medicine, University of Southern California, Los Angeles, California, United States of America; 3 Department of Preventive Medicine, Keck School of Medicine, University of Southern California, Los Angeles, California, United States of America; 4 Laboratory of Medicinal Chemistry, Center for Cancer Research, NCI-Frederick, Frederick, Maryland, United States of America; Sanger Institute, United Kingdom

## Abstract

Epigenetic modifications of the herpesviral genome play a key role in the transcriptional control of latent and lytic genes during a productive viral lifecycle. In this study, we describe for the first time a comprehensive genome-wide ChIP-on-Chip analysis of the chromatin associated with the Kaposi's sarcoma-associated herpesvirus (KSHV) genome during latency and lytic reactivation. Depending on the gene expression class, different combinations of activating [acetylated H3 (AcH3) and H3K4me3] and repressive [H3K9me3 and H3K27me3] histone modifications are associated with the viral latent genome, which changes upon reactivation in a manner that is correlated with their expression. Specifically, both the activating marks co-localize on the KSHV latent genome, as do the repressive marks. However, the activating and repressive histone modifications are mutually exclusive of each other on the bulk of the latent KSHV genome. The genomic region encoding the IE genes ORF50 and ORF48 possesses the features of a bivalent chromatin structure characterized by the concomitant presence of the activating H3K4me3 and the repressive H3K27me3 marks during latency, which rapidly changes upon reactivation with increasing AcH3 and H3K4me3 marks and decreasing H3K27me3. Furthermore, EZH2, the H3K27me3 histone methyltransferase of the Polycomb group proteins (PcG), colocalizes with the H3K27me3 mark on the entire KSHV genome during latency, whereas RTA-mediated reactivation induces EZH2 dissociation from the genomic regions encoding IE and E genes concurrent with decreasing H3K27me3 level and increasing IE/E lytic gene expression. Moreover, either the inhibition of EZH2 expression by a small molecule inhibitor DZNep and RNAi knockdown, or the expression of H3K27me3-specific histone demethylases apparently induced the KSHV lytic gene expression cascade. These data indicate that histone modifications associated with the KSHV latent genome are involved in the regulation of latency and ultimately in the control of the temporal and sequential expression of the lytic gene cascade. In addition, the PcG proteins play a critical role in the control of KSHV latency by maintaining a reversible heterochromatin on the KSHV lytic genes. Thus, the regulation of the spatial and temporal association of the PcG proteins with the KSHV genome may be crucial for propagating the KSHV lifecycle.

## Author Summary

KSHV is a ubiquitous herpesvirus that establishes a life-long persistent infection in humans and is associated with Kaposi's sarcoma and several lymphoid malignancies. During latency, the KSHV genome persists as a multicopy circular DNA assembled into nucleosomal structures. While viral latency is characterized by restricted viral gene expression, reactivation induces the lytic replication program and the expression of viral genes in defined sequential and temporal order. Posttranslational modifications of the viral chromatin structure have been implicated to regulate viral gene expressions but the underlying gene regulatory mechanisms are still elusive. Here, we demonstrate that the latent and lytic chromatins of KSHV are associated with a distinctive pattern of activating and repressive histone modifications whose distribution changes upon reactivation in an organized manner in correlation with the temporally ordered expression of viral lytic genes. Furthermore, we demonstrate that the evolutionarily conserved Polycomb group proteins, that maintain the repression of genes involved in hematopoiesis, X-chromosome inactivation, cell proliferation and stem cell differentiation, also play a critical role in the regulation of KSHV latency by maintaining a repressive chromatin structure. Thus, the epigenetic program of KSHV is at the crux of restricting latent gene expression and the orderly expression of lytic genes.

## Introduction

Chromatin is a highly dynamic structure of nucleosomes that are composed of DNA wrapped around the core histones (H2A, H2B, H3 and H4). Over the past decade, several studies have demonstrated that histones are subject to various posttranslational modifications (acetylation, methylation, phosphorylation, and ubiquitination), which are capable of modulating chromatin structures to thereby influence gene expression [1]. Hyperacetylation of histones H3 and H4 occurs mainly on promoters and correlates with gene activation, while hypoacetylation is characteristic of repressed genes [2]. Histone methylation is associated with either activation or repression of genes, depending on which histone lysine residues are mono-, di- or trimethylated. Various histone methylations are then recognized by specific chromodomain-containing proteins that can function as either transcription factors or as part of large chromatin remodelling/modifying complexes, which eventually determine the activity of target genes [1].

Histone methylation status fluctuates in response to environmental and developmental conditions. A number of enzymes that add or remove methylation modifications have been discovered [3], [4]. In general, transcriptionally active genes are associated with H3K4me3 and H3K36me3, whereas trimethylation of H3K9, H3K27 and H4K20 occurs primarily on repressed genes. H3K9me3 and H4K20me3 histone modifications are characteristic of pericentric heterochromatin, which is considered to be constitutive heterochromatin [5], [6], [7], [8], [9]. On the other hand, H3K27me3 is the marker of highly dynamic and reversible heterochromatin (facultative heterochromatin), and is characteristic of genes that are subject to tissue specific or developmentally regulated expression [10], [11], [12]. Genome-wide analysis of embryonic stem (ES) cells revealed that H3K27me3 preferentially localizes on developmental genes, which are repressed in stem cells but are expressed during ES cell differentiation [13], [14]. Interestingly, the promoter of large number of these developmental genes are also enriched in activating H3K4me3 suggesting that these genes are silenced but poised for rapid activation in ES cells [15]. Promoters enriched in both activating (H3K4me3) and repressive (H3K27me3) histone marks, called bivalent promoters, have been associated with rapidly inducible genes in T cells as well [7].

H3K27me3 is deposited by the evolutionary conserved 600-kDa Polycomb Repressive Complex 2 (PRC2), which consists of three Polycomb group (PcG) proteins (EZH2, SUZ12, EED) and the histone-binding proteins, RbAp48/46 [16]. The SET domain-containing EZH2 is an H3K27me3 histone methyltransferase, which can be found along entire genomic regions enriched with H3K27me3 in mammalian cells [17]. H3K27me3 provides a binding platform for PRC1, a larger Polycomb complex consisting of more than 10 subunits. In Drosophila, PcG proteins are recruited to their target genes via Polycomb response elements (PRE) that can be found in promoters [18]. It is still unclear how PcG proteins are recruited to their target genes in mammalian cells, but non-coding RNAs and specific DNA sequences similar to PREs have been implicated to be involved in this process. [16], [19], [20]. Polycomb-mediated gene silencing has been shown to be reversible with H3K27me3 demethylases such as JMJD3 and UTX, which can be recruited to the repressed promoters by transcription activators as has been shown, for instance, in the case of the H3K4me3 methyltransferase complexes [21], [22], [23], [24].

Viruses replicating in the nucleus are also under the influence of the chromatin during different stages of their life cycles. Therefore, viruses have evolved various mechanisms to utilize or neutralize the impact of cellular chromatin factors, to ultimately control viral replication and gene expression [25], [26], [27], [28]. Herpesviruses have a large DNA genome that persists as multicopy circular episomes associated with histones in the nucleus. Herpesviral infection can lead to two different life cycles: latency and lytic replication. During latency, the viral episomes are assembled into nucleosomal structures that resemble bulk cellular chromatin. Regulation of the chromatin structure of the Herpes simplex virus type 1 (HSV-1) genome has been implicated as the underlying cause of the switch between latency and lytic replication as well as being involved in the regulation of lytic gene expression [29], [30].

A human tumour virus, called Kaposi's sarcoma-associated herpesvirus (KSHV) or human herpesvirus 8 (HHV8), has been consistently identified in Kaposi's sarcoma (KS) tumours, pleural effusion lymphoma (PEL), and Multicentric Castleman disease [31], [32], [33]. Several studies have indicated that DNA methylation and histone acetylation can play a role in the regulation of KSHV gene expression [34], [35]. The immediate early KSHV gene encoded by ORF50, called RTA (replication and transcription activator), is the master regulatory factor and is sufficient to induce the complete cycle of viral replication [36], [37]. The RTA promoter associates with histone deacetylases (HDACs) during latency resulting in hypoacetylated histones [34]. Treatment of latently infected KSHV positive cells with HDAC inhibitors, butyrate or TSA, induces the hyperacetylation of viral chromatin concomitantly with the recruitment of histone acetyltransferases, chromatin remodelling proteins (Ini1/Snf5) and the TRAP/Mediator coactivator complex to the RTA promoter, allowing RTA expression to induce the complete viral gene expression cascade [34], [38].

To elucidate the characteristics of the KSHV epigenome, we performed a high-resolution genome-wide analysis to map a set of activating and repressive histone H3 modifications on the entire KSHV genome during latency and reactivation. Based on their genome-wide profiles, we found that the KSHV genes are associated with a distinctive pattern of active and repressive histone modifications during latency, which ultimately changes upon reactivation. Importantly, the promoter regions of RTA and several E genes are associated with both H3K4me3 and H3K27me3 marks, suggesting that these promoters have a bivalent chromatin structure that maintains their repression during latency and is also poised for rapid activation upon stimulation. We also found that while the EZH2 histone methyltransferase is colocalized with H3K27me3 on the entire KSHV latent genome, it rapidly dissociates from the RTA promoter and other IE-E gene-rich genomic regions upon reactivation. This event ultimately results in reduced level of H3K27me3, which are concomitant with increasing levels of activating histone marks on the RTA promoter. Furthermore, treatment of latently KSHV-infected cells with a drug inhibiting the expression of PcG proteins, the small inhibitory RNA-mediated knockdown of EZH2 or the overexpression of H3K27me3 histone demethylases efficiently trigger the lytic reactivation of KSHV. These data collectively demonstrate that the Polycomb group proteins are involved in the maintenance of KSHV latency by preserving a reversible heterochromatin on the promoter regions of lytic genes such that they are silenced during latency but are poised for rapid activation upon reactivation.

## Results

### Dissociation of histone H3 from the KSHV genome is concomitant with viral DNA replication

In this study we asked what histone modifications are associated with the KSHV genome during latency and how they change upon reactivation. For this, we used the well-characterized recombinant KSHV-positive primary effusion lymphoma cell line, TRExBCBL1-RTA, which expresses a Doxycycline (Dox) inducible myc/His-tagged RTA incorporated into the cellular genome [39]. We chose the RTA-mediated reactivation of KSHV instead of chemical inducers such as TPA, TSA or sodium butyrate because these chemicals can globally affect both cellular and viral gene expression, while RTA ensures robust and specific viral reactivation [39]. Figure S1 showed that Dox treatment (1ug/ml) of the TRExBCBL1-RTA cells for 6, 12 and 24 hours rapidly induced myc/His-RTA expression, resulting in a gradual induction of the KSHV gene expression cascade (Figure S1A, B) [39], whereas viral DNA replication was apparent only at 24 hpi, which was in correlation with the induction of late gene expressions (Figure S1B and C). To analyse the changes in the KSHV nucleosome structure upon reactivation, ChIP experiments were performed with a histone H3 specific antibody. We measured the abundance of specific DNA sequences in the histone H3 immunoprecipitates with qPCR using a set of primer pairs specific for both the promoter and the coding regions of various viral genes including RTA, LANA, K2, ORF8, ORF25, ORF56 and ORF64 (Figure S2A, B). This revealed comparable levels of H3 association throughout the viral genome during latency (0 hpi), which did not significantly change on most of the genomic regions at 12 hpi, but dropped sharply at 24 hpi (Figure S2B). This is in agreement with previous findings showing that disassembly of the viral chromatin is concomitant with viral DNA replication during lytic infection [40]. In contrast, H3 levels remained constant on cellular promoters during KSHV lytic reactivation, suggesting that H3 dissociation occurs specifically on the viral genome (Figure S2C).

### Distinct patterns of activating and repressive histone modifications on the KSHV genome during latency and reactivation

**Figure 1:**
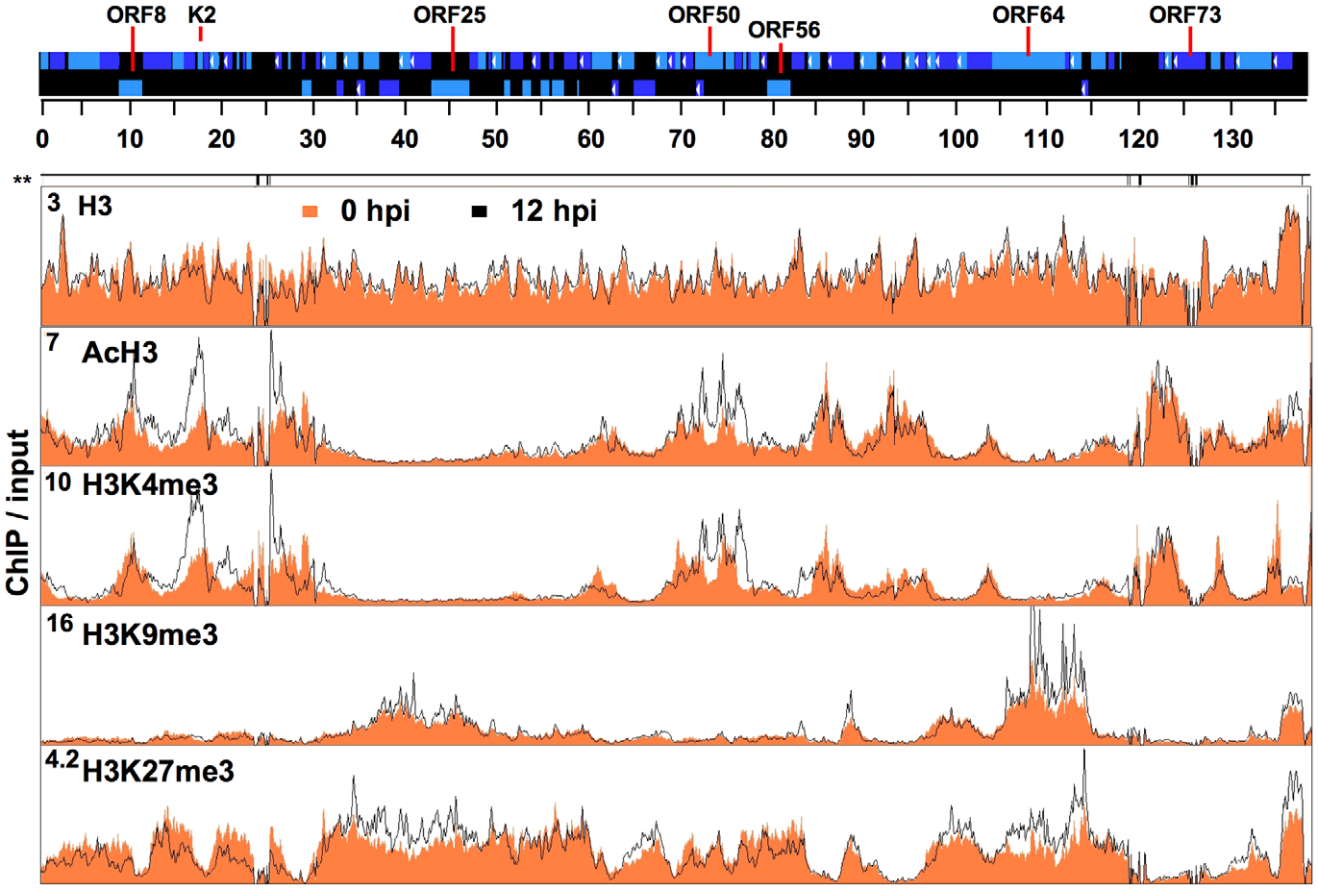
Genome-wide mapping of histone modifications on the KSHV genome during latency and reactivation. Each ChIP-on-chip experiment is an average of two biological replicates. The histone H3 and histone modification ChIPs were performed with non-induced and doxycycline-induced (12 hpi) TRExBCBL1-Rta cells followed by the hybridization of the labelled ChIP and input DNAs onto a custom designed KSHV-specific 15-bp tiling microarray. See the Material and methods for details. Orange colour indicates 0 hpi-ChIP/input ratio while the black line shows 12 hpi-ChIP/input. Numbers in the left upper corners show the maximum values of Cy5/Cy3. Missing probes in specific genomic regions are shown below the genome scale (**). The alternating dark and light blue squares atop display the viral ORFs where the white triangle indicates ORFs that are expressed from the reverse DNA strand. The “hpi” stands for hours post-induction.

In order to investigate the epigenome of KSHV during latency and lytic reactivation, we tested whether repressive histone modifications associated with lytic genes are responsible for maintaining the repression of lytic gene expression during latency and whether reactivation induces the deposition of activating histone modifications onto the viral genome for lytic gene expression. To uncover the global distribution of histone modifications on the KSHV chromatin during latency and reactivation, we mapped the genome-wide distribution of activating histone marks [acetyl-H3 (AcH3) and H3K4me3] and repressive marks (H3K9me3 and H3K27me3) on the KSHV genome (Figure 1 and Figure S3, S4, S5, S7). The ChIP-on-chip experiments were carried out with chromatins prepared from both non-induced (0 hpi/latency) and Dox-induced (12 hpi) TRExBCBL1-RTA cells. Figure 1 shows the average of two independent ChIP-on-chip biological replicates (Figure S3 and S4). To get a high resolution of the localization of histone modifications and proteins of interest on the KSHV genome, we used a 15-bp tiling microarray that contains 8942 overlapping 60 nucleotide-long oligos spanning the entire KSHV genome.

Because changes in H3 occupancy may affect the enrichment of histone modifications on the viral genome, we first investigated the global distribution of H3 on the viral genome during latency and upon reactivation. The histone H3 ChIP-on-chips revealed that histone H3 enrichment levels were comparable throughout the KSHV genome at 0 hpi and did not significantly change on most parts of the KSHV genome at 12 hpi (Figure 1, S3, S4). The histone H3 ChIP analysis also showed similar results (Figure S2B). However, it should be noted that a small viral genomic region between 15 and 30 kb, which contains mostly KSHV unique genes, displayed a detectable decrease of H3 occupancy at 12 hpi (Figure 1). In contrast to the uniform distribution of H3, the different histone modifications displayed distinct patterns and were enriched in specific KSHV genomic regions during latency and reactivation (Figure 1). Immunoblot analysis revealed that the expression of H3 as well as the global levels of cellular histone modifications did not change upon KSHV reactivation (Figure S2 D), thus any change of the histone modifications on the KSHV genome is likely to be a consequence of the reactivation-induced change in the KSHV epigenome. The genome-wide mapping of histone modifications showed that both activating and repressive histone modifications were associated with the KSHV genome during latency (Figure 1). While both the activating AcH3 and H3K4me3 marks co-localized on the KSHV genome so did the repressive H3K9me3 and H3K27me3 marks. However, the activating and repressive histone modifications were mutually exclusive on the bulk of the KSHV genome (e.g. 30–60 kb and 90–120 kb) (Figure 1). As expected, the latency-associated locus (118–128 kb) where KSHV genes that are constitutively expressed during latency are located, was enriched with the activating H3K4me3 and AcH3 histone modifications but depleted for the repressive H3K9me3 and H3K27me3 (Figure 1). Unexpectedly, we also found that several regions of the KSHV genome (e.g. 1–30 kb and 60–90 kb) that are not associated with latency-gene expression were also highly enriched in both H3K4me3 and AcH3 histone marks (Figure 1). Strikingly, the PcG protein-mediated repressive histone modification, H3K27me3, was widely distributed throughout the KSHV genome, whereas the H3K9me3 repressive histone modification was restricted mainly to two genomic regions (30–60 kb and 95–115 kb) encoding a number of late genes (Figure 1). Interestingly, the activating H3K4me3 and AcH3 histone modifications were absent in these two genomic regions where the repressive H3K9me3 and H3K27me3 histone modifications coexisted, suggesting that KSHV genes in these regions have a strongly repressive heterochromatin structure during latency (Figure 1).

The RTA-induced initiation of KSHV lytic gene expression program results in the redistribution of histone modifications on viral genome. We calculated the 12hpi/input ratio to view the changes in histone modifications (Figure 1). Since H3 enrichment was comparable between 0 and 12 hpi on most parts of the viral genome and viral DNA replication had yet to occur by 12 hpi, we also hybridized the 12 hpi-ChIPs against the 0 hpi-ChIPs, which allowed us to specifically observe changes in histone modification levels on the KSHV genome upon lytic reactivation (Figure S7C). Others have also recently applied similar ChIP-on-chip analyses in studies investigating the epigenetic reprogramming of the host genome by the adenoviral protein E1a [41]. Our ChIP-on-chip analysis showed that the enrichment of the activating histone modifications was elevated the most when it was concomitant with the reduction of repressive histone marks in the genomic regions between 1 and 30 kb containing several early genes and between 68 and 77 kb, which encodes the IE proteins ORF45, ORF48, ORF50 (RTA) and K8 (KbZIP) (Figure 1 and S7). These changes in the viral chromatin are indicative of robust transcriptional activation and are consistent with the KSHV gene expression profile in that expression of the lytic genes in these genomic regions is induced in the early phase of reactivation [39], [42], [43]. In contrast, only minor changes in the levels of the AcH3 and H3K4me3 activating marks were detected in the genomic regions that encode large number of late genes (30–60 kb and 95–115 kb), whereas significant changes of the H3K27me3 repressive mark were observed at 12 hpi (Figure 1 and S7).

In summary, these results demonstrate that the activating histone modifications of the latent KSHV genome are preferentially enriched in the constitutively active latency-associated genomic locus and in the early-lytic gene-containing genomic regions. In contrast, the H3K9me3 and H3K27me3 repressive histone marks are primarily enriched in the genomic regions that encode many late genes during latency (0 hpi), and they remain associated with these regions during the early phase (12 hpi) of reactivation as well.

### Profiling the histone modification patterns within the regulatory regions of KSHV genes

**Figure 2:**
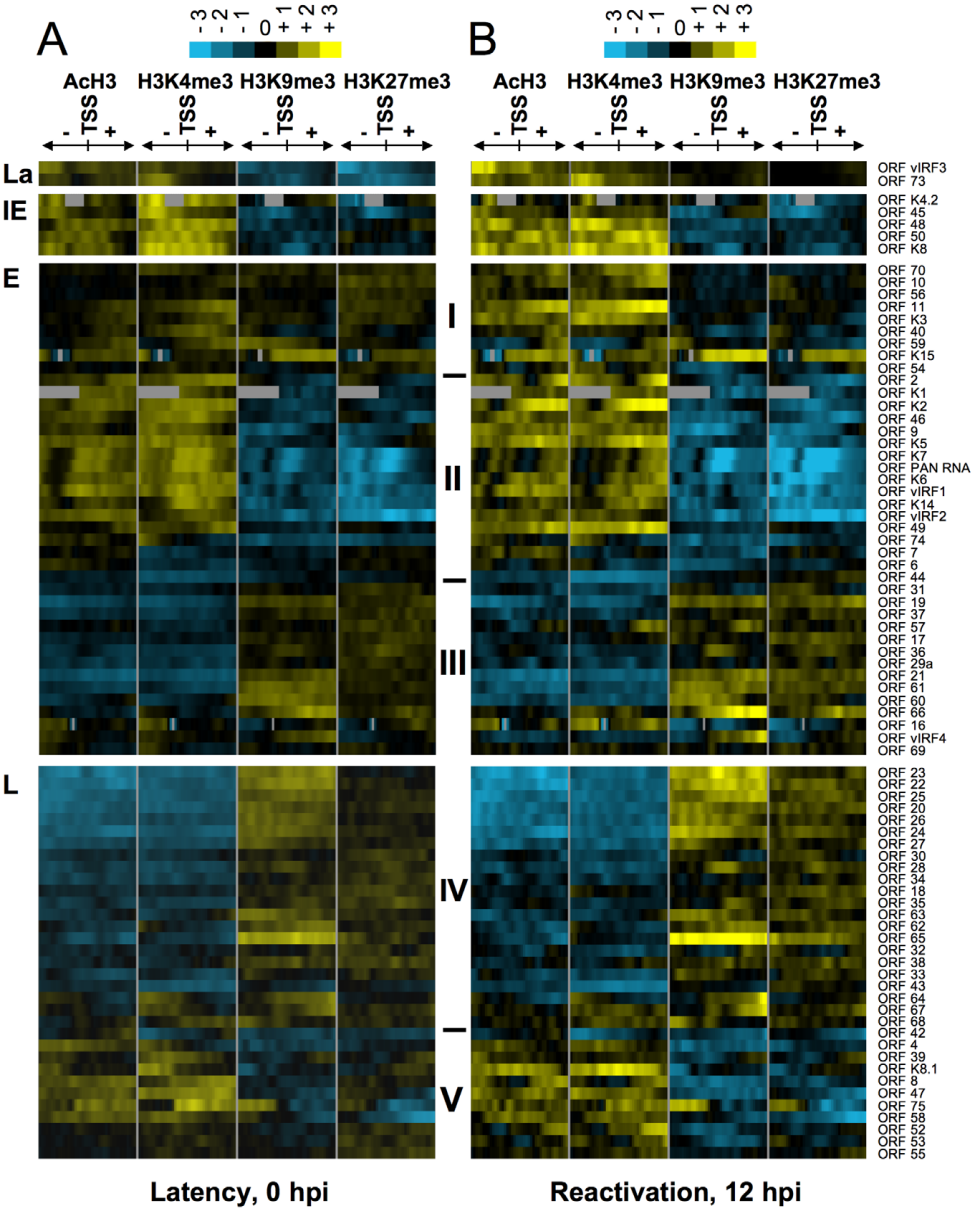
Hierarchical clustering of histone modifications associated with the regulatory regions of viral genes. Based on their expression patterns the viral genes were grouped as latent (La), IE, E and L genes and hierarchical clustering was performed within the groups. The rows display the histone modification patterns along the −1 kb to +1 kb genomic regions relative to the translational start site (TSS) of each viral gene, which we assigned for the gene regulatory regions. The 1 kb regions are divided into twenty 50 bp fragments that show the average of log2 ratio of probe signal intensities derived from the average of the biological replicates of ChIP-on-chip experiments. Blue and yellow colours represent lower-than-average and higher-than-average for enrichment, respectively, whereas gray shows missing values for enrichment due to lack of probes in those genomic regions. I-V represents the clusters of genes that have similar histone modification patterns. (**A**) Distinctive histone modification patterns are associated with the KSHV genes of different expression classes during latency. (**B**) Changes in the enrichment of histone modifications during reactivation (12hpi).

While only a few genes are expressed during latency, all viral genes are expressed upon lytic reactivation in a temporal and sequential order. This suggests that distinctive histone modifications may be associated with the different viral promoters to ultimately determine the timing and rate of viral gene expression. Thus, we attempted to delineate the characteristics of the chromatin structures associated with the regulatory regions of KSHV genes during latency and lytic reactivation (Figure 2, S6, S8). For this, we aligned the KSHV open reading frames (ORFs) relative to their translational start sites (TSS) and plotted the signal intensities of probes derived from the ChIP-on-chip analysis across a 2-kb region spanning 1 kb on either side the TSS (please see Materials and Methods for details). The rationale of this strategy was based on a number of considerations. (i) While the transcriptional start sites have only been identified for a few KSHV genes, they are generally within a few hundred base pairs of their TSS, showing that the TSS can be used as a reference point. (ii) Due to the compact structure of the KSHV genome, the promoters are usually closely localized upstream of the TSS. (iii) The 1 kb downstream region of the TSS was included in the analysis because several KSHV genes have introns close to the TSS at their 5′ ends, which may contain gene regulatory elements. Based on these factors, the 2-kb sequences around the TSS were considered to be the gene regulatory regions of KSHV genes. In fact, a similar strategy has also been used to analyse the recruitment of the KSHV transactivators, Rta and K-bZIP, to eighty-three putative KSHV promoters in TRExBCBL1-RTA cells [44]. Using the signal intensities of the probes derived from the ChIP-on-chip analysis, we performed an average linkage hierarchical clustering within each gene class (La or latent, IE, E, L), which gave us a comprehensive overview of the repressive and activating histone modifications across all the 2-kb TSS regions (Figure 2 and S8).

#### IE gene class

**Figure 3:**
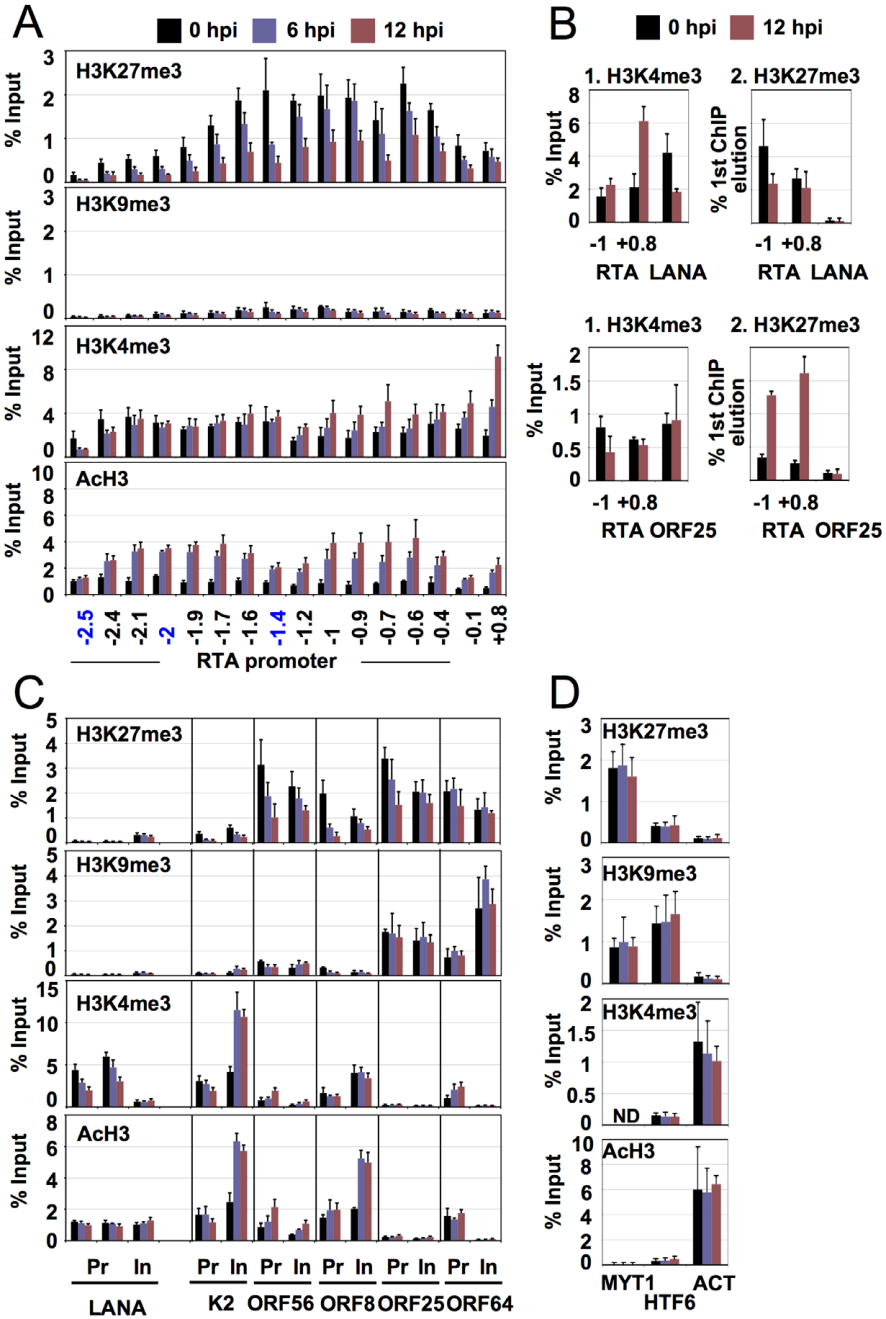
Dynamic association of histone modifications with viral genes during latency and reactivation. (**A**) Time-course ChIP analysis of histone modifications on the RTA promoter during latency and reactivation. Cellular controls can be seen in panel D. (**B**) Colocalization of H3K4me3 and H3K27me3 on the RTA promoter is confirmed by sequential ChIP assays. The first ChIP was performed with either H3K4me3-specific or H3K27me3-specific antibody, followed by the elution of the immunoprecipitated DNAs and a second ChIP with either H3K27me3 or H3K4me3 antibody. LANA and ORF25 promoters were used as controls. (**C**) Time-course ChIP analysis of histone modifications on the selected latent (LANA), early (K2, ORF56) and late (ORFs 8, 25, 64) genes. Cellular controls can be found in panel D. Pr: promoter, in: within gene body. (**D**) ChIP assays of histone modifications on cellular promoters. The promoters of the repressed cellular MYT1 and HTF6 genes as well as the active promoter of the actin (ACT) gene were also tested using the same ChIP samples that had been used in panels A and C. ND: not detectable.

By definition, IE genes are the first set of expressed viral genes whose induction does not require *de novo* expression of any viral proteins and can be rapidly induced upon reactivation of KSHV from latency. We found that although the IE genes (K4.2, K8, ORF45, ORF48, ORF50/RTA) are silenced during latency, their 2-kb regulatory regions are enriched in the activating histone modifications H3K4me3 and AcH3, and this is further increased upon lytic reactivation at ORFs 48, 50 and K8 (Figure 2, S8, S9). Since RTA is responsible for the switch between latency and lytic replication, not only is its promoter tightly repressed during latency, but its silencing should also be rapidly reversible upon reactivation. We found that the RTA promoter is enriched in both H3K4me3 and H3K27me3 during latency, suggesting that it possesses a bivalent chromatin that maintains the repression of the RTA promoter while keeping it poised for rapid activation (Figure 2, S8). An extensive ChIP analysis further confirmed the enrichment of AcH3, H3K4me3 and H3K27me3 and the depletion of H3K9me3 on the 2.5-kb promoter region of RTA during latency (Figure 3A). Upon lytic reactivation, however, the repressive H3K27me3 gradually decreased, while the activating histone modifications H3K4me3 and AcH3 increased on the RTA promoter (Figure 3A). These changes in the histone modification pattern are in concert with the induction of RTA expression.

To confirm that the changes in the enrichment of AcH3, H3K4me3 and H3K27me3 were not due to differences in the efficiency of ChIPs between 0 and 12 hpi and that the low level of H3K9me3 was not due to a low efficiency of the H3K9me3-ChIP, three cellular promoters were also included as controls. Figure 3D shows that the levels of histone modifications on the transcriptionally repressed promoters of MYT1 and HTF6 genes as well as on the transcriptionally active promoter of the actin (ACT) gene did not significantly change between 0 hpi and 12 hpi, indicating that the efficiencies of the ChIPs were comparable at different time points (Figure 3D).

#### Bivalent chromatin on the RTA promoter during latency

Although >95% of non-induced TRExBCBL1-RTA cells were in latency, a few percentages of the cells spontaneously underwent lytic reactivation as measured by the surface expression of the early viral protein ORF K1 in flow cytometry analysis (data not shown). Thus, this raises the possibility that the colocalization of H3K4me3 and H3K27me3 histone modifications at the same genomic region may be due to the mixture of latent and spontaneously reactivated KSHV genomes in the ChIPs assays. To determine whether H3K4m3 and H3K27me3 on the RTA promoter were simultaneously on the same KSHV genome and not independent histone marks on different viral genomes, sequential ChIP assay was applied (Figure 3B). We performed the first ChIP with an anti-H3K4me3 antibody, followed by the elution of the immunoprecipitated chromatin, which was then used in a second ChIP with an anti-H3K27me3 antibody. The ChIP DNAs were measured by qPCR, using specific primers for the promoter regions of RTA, LANA and ORF25 genes. This showed that both H3K4me3 and H3K27me3 were enriched only on the RTA promoter (Figure 3B, top panel). The reciprocal order of the ChIPs gave the same results (Figure 3B, bottom panel). The promoter regions of LANA and ORF25 were included as controls and showed the presence of either H3K4me3 or H3K27me3, respectively, but not both (Figure 3B). These data collectively show that the H3K4me3 and H3K27me3 associated with the RTA promoter coexist on the same KSHV genome, indicating that the RTA promoter appears to possess a bivalent chromatin.

#### Early gene class

Expression of IE genes is followed by the induction of E genes that encode a large variety of proteins for modulating immune responses, apoptosis or performing viral DNA synthesis. From the analysis of the TSS/promoter regions of E genes, three subgroups (I, II and III) of histone modification patterns emerged (Figure 2, S8). Group I: some of the E genes also seem to have a bivalent chromatin structure on their 2-kb putative regulatory regions during latency. Upon reactivation, this changes to fully active euchromatin with increased activating and decreased repressive histone modifications on their promoter (Figure 2, S8). Group II: the promoter regions of the majority of E genes are associated only with AcH3 and H3K4me3 but depleted for the repressive marks during latency (Figure 2, S8). Interestingly, groups I and II consist of a number of E genes that are involved in the deregulation of host immune response (K1/3/5/15, ORFs 10, 11) and/or KSHV pathogenesis (K2/4.1/6/9, ORF74) (Figure 2, S8). Group III: this group represents a cluster of E genes that are enriched in H3K27me3 and H3K9me3 but depleted for activating histone modifications during latency, which does not change significantly upon reactivation. It should be noted that the histone modification pattern of Group III genes resembles that of Group IV late genes, even though their expression patterns are considerably different during KSHV lifecycle. The above findings were further confirmed in independent ChIP experiments analysed by qPCR using specific primers for the K2 and ORF56 promoter and coding regions (Figure 3C).

#### Late gene class

The KSHV genes whose expression can be blocked by viral DNA replication inhibitors are classified as late genes. Because most of the late gene products are necessary for the assembly and egress of viral particles, their expression is required only after viral DNA synthesis. To achieve this temporal order of viral gene expression they should be completely silenced during the expression of IE and E genes. In agreement with this notion, we found that the promoter regions of most of the late genes were enriched in the repressive H3K27me3 and H3K9me3 histone modifications and depleted in the activating histone marks during latency and in the early phase of the reactivation (12 hpi) (Figure 2, S8, group IV). In contrast, the TSS regions of some late genes (e.g. ORFs 39, 8, 47, 75) are associated with high levels of activating and low levels of repressive histone modifications during latency, which slightly changed during reactivation (Figure 2, S8, group V). It is also worth noting the difference in location between groups IV and V late genes on the KSHV genome, which may explain their distinct chromatin structures. The group IV late genes, which are highly enriched in repressive histone modifications, are clustered in the 30–60 kb and 95–115 kb genomic regions (see Figure 1B). On the other hand, the few L genes in the group V are scattered throughout genomic regions enriched with activating histone modifications. These results were further confirmed by a series of ChIP experiments of the promoter and coding regions of three late genes: ORF8 (group V), ORF25 (group IV, 30–60 kb) and ORF64 (group IV, 95–115 kb) (Figure 3C). These observations suggest that late genes can be regulated by distinctive mechanisms depending on their location on the KSHV genome.

#### Latent gene class

In agreement with the observed constitutive expression of the latent genes (LANA and vIRF3) during latency, their 2-kb TSS regions display enrichment only in H3K4me3 and AcH3 while lacking repressive histone modifications (Figure 2). Interestingly, while H3K4me3 enrichment decreased somewhat, AcH3 levels remained constant on the LANA promoter upon reactivation (Figure 3C). This was also confirmed by additional independent ChIP experiments with qPCR analysis focusing on the specific locations within the LANA promoter and coding regions (Figure 3C).

In summary, we described the chromatin modification patterns of the TSS regions of the four expression classes (Latent, IE, E, and L) of KSHV genes during latency and lytic reactivation. Based on these data we propose that the diversity of the chromatin structure of the KSHV genome can reflect the various regulation mechanisms of viral gene expression, which allows a temporal and well-organized gene expression during reactivation.

### Dynamic association of the Polycomb group proteins and transcriptional activators with the KSHV genome during latency and lytic reactivation

**Figure 4:**
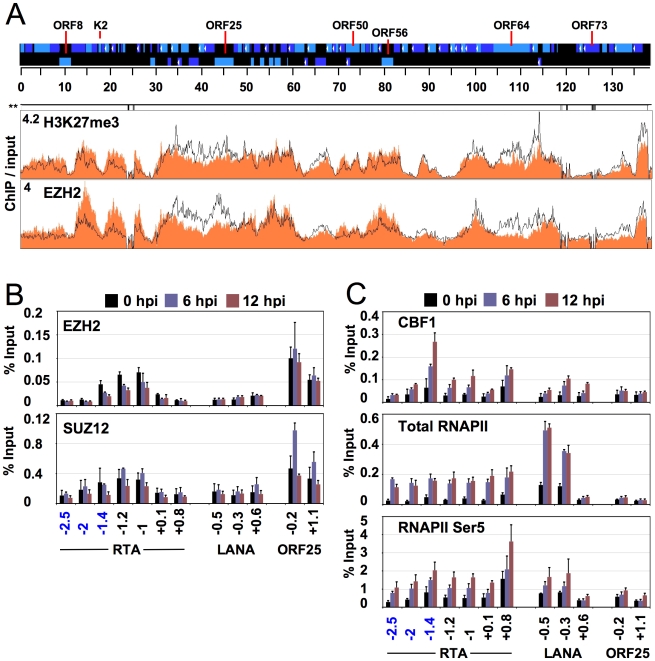
Genome-wide binding of EZH2 to the KSHV genome correlates with the repression of lytic genes. (**A**) ChIP-on-chip was performed for EZH2 and its genome-wide binding was compared with the distribution of H3K27me3 on the KSHV genome during both latency and reactivation. Labels are the same as in Figure 1. The H3K27me3 graph was taken from Figure 1. (**B**) EZH2 and SUZ12 binding to the H3K27me3-rich lytic promoters are shown by independent ChIP assays. EZH2-interacting PcG protein SUZ12 is enriched only where EZH2 is present. (**C**) Recruitment of transcription activators to the activated lytic promoters during KSHV reactivation. An anti-RNA polymerase II antibody (H-224) that recognizes the RNAPII independently from its phosphorylation state (total RNAPII) was used for ChIP of total RNAPII, while the anti-RNA polymerase II antibody CTD4H8 specifically immunoprecipitates RNAPII phosphorylated at the 5^th^ serine of its C-terminal domain (RNAPII Ser5).

Because of the genome-wide distribution of H3K27me3 on the KSHV genome, we performed a ChIP-on-chip assay to determine whether EZH2, the H3K27me3 histone methyltransferase of the PcG proteins, associates with the KSHV genome. (Figure 4A). ChIP-on-chip assays showed that EZH2 almost completely colocalized with H3K27me3 throughout the entire KSHV genome during both latency and reactivation (Figure 4A, S7, S10). Additional ChIP assays showed that EZH2 and SUZ12 (another subunit of the PcG complex PRC2) were found on the RTA and ORF25 promoters enriched with H3K27me3, but not on the LANA promoter (Figure 4B).

Besides its regulatory role in cellular gene expression, the CBF1 transcription activator has been shown to play an active role in KSHV gene expression as well [45], [46]. Since the RTA promoter contains several CBF1 binding sites (Figure S11 A) [47], [48], we studied the recruitment of CBF1 onto the RTA promoter during reactivation. The ChIP assay showed that CBF1 was recruited to the RTA promoter, primarily at 1.4 kb upstream of the RTA translational start site where three putative CBF1 binding sites are closely located (Figure 4C and Figure S11 A). Furthermore, not only does this putative CBF-binding region of RTA promoter efficiently binds CBF1 *in vitro*, but its deletion also resulted in a dramatic decrease of the RTA-mediated autoactivation of its own promoter (Figure S11 B, C). Additional ChIP experiments revealed that RNAPII is also recruited to the promoter regions of RTA and LANA upon reactivation (Figure 4C). Recruitments of CBF and RNAPII to the RTA and LANA promoters were specific since they were not recruited to the promoter of the late gene, ORF25, whose expression was still blocked at 6 and 12 hpi (Figure 4C and S1B). The increase of RNAPII over the 3-kb upstream region of RTA may be not surprising given that this genomic region also includes the promoters of other lytic genes (ORFs 45, 46, 47, 48) as well as an alternative upstream promoter for RTA [49]. These results illustrate the dynamic associations of the PcG complex and transcriptional activators with KSHV genome during latency and lytic reactivation.

### Polycomb group protein EZH2 and its H3K27me3 histone mark are required for the maintenance of KSHV latency

**Figure 5:**
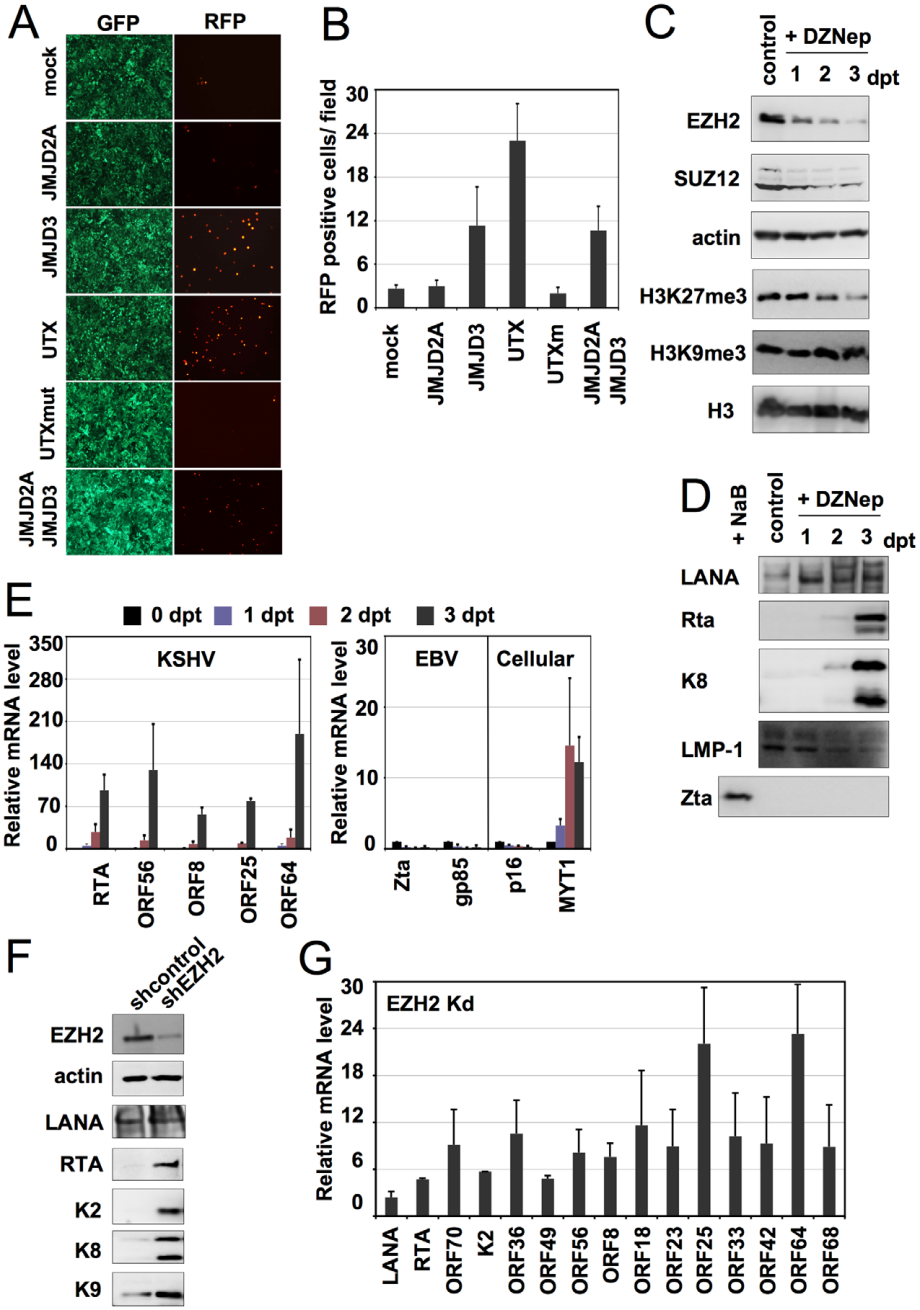
Polycomb group proteins are involved in the maintenance of latency of KSHV. (**A**) Overexpression of the wild-type HA-tagged H3K27me3 histone methyltransferases (HMTs) UTX and JMJD3 triggers the lytic reactivation of KSHV in Vero-rKSHV.219 as shown with the expression of RFP. In contrast, the H3K9me3 HMT JMJD2A and the enzymatically inactive UTXm showed little or no effect on KSHV lytic reactivation. (**B**) Quantification of RFP positive cells. (**C** and **D**) JSC-1 cells were treated with 5 uM DZNep for 1, 2 and 3 days and the cells were harvested for immunoblot analysis with the indicated specific antibodies against cellular proteins and histone modifications (C) or viral proteins (D). “Dpt” indicates days post-treatment. Whole cell lysate of NaB-treated JSC-1 cells was used as a control for Zta immunoblot. (**E**) JSC-1 cells were treated with DZNep as described in (C) and total RNAs were isolated for RT-qPCR analysis of some selected KSHV, EBV and cellular mRNAs. (**F** and **G**) BCBL-1 cells were infected by lentivirus expressing the indicated shRNAs and were then subject to immunoblotting analysis with the indicated antibodies (F) or RT-qPCR analysis was performed for the indicated viral transcripts (G).

The dynamic association of EZH2 with KSHV genome suggests that PcG-mediated H3K27me3 histone modification is involved in the repression of lytic gene expression during latency. To address this issue, HA-tagged JMJD2A, JMJD3 and UTX histone demethylases were expressed in Vero-rKSHV.219 cells to test if the elimination of histone methylations can trigger KSHV reactivation (Figure 5A). JMJD2A is an H3K9me3-specific histone demethylase [50], JMJD3 and UTX are H3K27me3-specific histone demethylases [21], [51], and UTXmut is an enzymatically inactive form of UTX that contains a single point mutation (H1146A) in the Fe^2++^ ion binding site [51]. JMJD3 and UTX have been shown to eradicate the H3K27me3 repressive mark, resulting in the upregulation of PcG-targeted gene expression [21], [52]. JMJD2A and JMJD3 or UTX expression detectably suppressed the steady-state levels of H3K9me3 or H3K27me3, respectively, in transfected Vero cells (Figure S12B, C). Since Vero-rKSHV.219 cells express red fluorescent protein (RFP) from the KSHV lytic PAN promoter and green fluorescent protein (GFP) from the EF-1α promoter, RFP expression has been extensively used as a marker of KSHV lytic reactivation [53]. Immunofluorescence analysis revealed that JMJD3 and UTX efficiently triggered KSHV reactivation, while JMJD2A and UTXmut did not (Figure 5A, B). Furthermore, coexpression of JMJD2A and JMJD3 showed no significant synergistic effect on KSHV reactivation (Figure 5A, B). Finally, JMJD2A, JMJD3, UTX and UTXmut were expressed at comparable levels (Figure S12A). These results bespeak the importance of the H3K27me3 histone modification in the maintenance of KSHV latency.

A small molecule, 3-Deazaneplanocin A (DZNep), has been shown to inhibit the expression of the Polycomb repressive complex 2 (PRC2) components (EZH2, SUZ12, and EED), resulting in the suppression of H3K27me3 histone methylation and the upregulation of PcG target genes *in vivo* [54]. To further test the role of H3K27me3 histone methylation in KSHV latency, we treated KSHV and EBV co-infected primary effusion lymphoma JSC-1 cell line with DZNep, followed by immunoblotting assays (Figure 5 C and D). DZNep treatment dramatically decreased the EZH2 and SUZ12 and, thereby, H3K27me3 levels, ultimately resulting in the induction of the expression of polycomb-targeted cellular MYT1 gene (Figure 5E). However, H3K9me3, histone H3, and actin levels were not affected under the same conditions (Figure 5C). We found that DZNep treatment efficiently induced the reactivation of KSHV, but not EBV: KSHV Rta and K8 expressions were detected as early as 2 days after DZNep treatment, while the EBV IE protein Zta was not induced (Figure 5D). Real time quantitative RT-PCR also showed the induction of other early (ORF56) and late KSHV genes (ORFs 8, 25, 64), suggesting that PRC2 depletion activates the gene expression cascade of KSHV from the repressed latent state (Figure 5E).

Besides the downregulation of EZH2 and SUZ12, DZNep also induced apoptosis of JSC-1 cells, detected by monitoring the cleavage of the apoptosis marker PARP (Figure S13 A, B). To exclude the possibility that apoptosis may have influenced KSHV reactivation, we treated JSC-1 with stauorosporine (STS), which also induced apoptosis in JSC-1 as shown by the cleavage of PARP (Figure S13 C). In contrast to DZNep treatment, STS treatment affected neither the steady state levels of EZH2 and H3K27me3, nor did it reactivate KSHV from latency (Figure S13 C, D). This suggests that DZNep-mediated H3K27me3 reduction triggers KSHV reactivation. This was further supported by the fact that specific shRNA-mediated depletion of EZH2 induced the expression of number of lytic genes (Figure 5F, G). In summary, the depletion of the PcG proteins in latently infected cells induces the lytic reactivation of KSHV, suggesting that PcG proteins play an important role in the maintenance of KSHV latency.

## Discussion

The genome-wide transcriptional analysis of KSHV gene expression revealed that despite the differences in the features of their promoters, viral genes with similar functions display analogous expression patterns during the lytic replication cycle, implying the existence of a common regulatory mechanism for their gene expression [39], [42], [43], [55]. In fact, cellular genes with related functions often have common chromatin structures that are associated with specific histone modifications whereby expression of large sets of genes can be coherently coordinated by epigenetic mechanisms [56]. Our ChIP-on-chip analysis identifies several distinct chromatin domains with different histone modifications on the latent KSHV genome, suggesting that expression of viral genes within each chromatin domain may be co-regulated (Figure 1, 2, S7, S8). Specifically, latent genes clustered in the latency-associated genomic locus have H3K4me3/AcH3-rich chromatin domain during latency and reactivation, which is in correlation with the constitutively active transcription of latency-associated genes. The genomic region encoding the IE genes ORF50 and ORF48 has a bivalent chromatin domain defined by the concomitant presence of the activating H3K4me3 and the repressive H3K27me3 marks during latency, which rapidly changes upon reactivation with increasing AcH3 and H3K4me3 and decreasing H3K27me3 (Figure 2, 3A). Importantly, the chromatin bivalency of the RTA promoter ensures the repression of RTA during latency, but also readies it for rapid activation upon reactivation (Figure 3A, S1). Bivalent chromatin is characteristic of many inducible cellular genes that are involved in the regulation of development and immune responses such that these genes are repressed yet primed for rapid activation [7], [15].

KSHV genomic regions encoding a number of late genes are associated with repressive H3K9me3 and H3K27me3 modifications during both latency and the early phase of lytic reactivation, which is in accordance with the observed silencing of late genes. Strikingly, the high expression of late genes is observed at the time of the viral DNA replication concomitantly with the disassembly of viral chromatin (Figure S1, S2). These data suggest that viral DNA replication may play a role in the disruption of the repressive heterochromatin associated with viral late genes, which ultimately facilitates late gene expression [40]. However, a recent study has shown that a portion of replicating herpesviral genomes can be chromatinized even during lytic replication, indicating that viral chromatin may be continuously involved in the regulation of late gene expression [57]. In addition, the deletion of several MHV68 genes has been shown to result in blocking of late gene expressions without affecting viral DNA replication [58], [59], [60]. This suggests that viral replication may not directly influence the disassembly of the heterochromatin of late genes.

Based on the histone modification patterns associated with the promoter regions of E genes, three distinctive groups could be observed (Figure 2, S8). The chromatin structure of the promoters of Group I genes resembles that of cellular bivalent promoters with the exception that they are also enriched in H3K9me3 during latency. However, the depletion of H3K9me3 is contemporaneous with the decrease of H3K27me3 upon reactivation, suggesting that both repressive histone marks are replaced by activating histone marks upon reactivation. The promoters of Group II genes are enriched primarily with activating histone modifications during both latency and reactivation, similar to those of IE genes, whereas the promoters of most Group III genes are mainly associated with repressive histone marks during latency and in the early phase of reactivation (12 hpi), resembling Group IV late genes. This indicates that despite the different gene expression profiles during the lytic reactivation cycle, some of the lytic genes show similar histone modification patterns in their promoters, suggesting that other histone modifications may be also associated with these promoter regions to generate the distinct gene expression profiles. This topic will be actively investigated in the future. Furthermore, our observation that the TSS regions/promoters of several E genes are enriched with the activating AcH3 and H3K4me3 histone marks but depleted for the repressive histone modifications during latency, suggests that although transcription of these E genes may have been initiated, RNAPII is likely stalled on their promoters (Figure 2 and 3C). In fact, several of these E genes (K2, K5, K6, K7, K11) have been shown to be temporally expressed immediately after *de novo* infection or rapidly express upon lytic reactivation [55], [61]. These E genes carry immune modulatory and/or antiapoptotic functions so their rapid expressions seem to be crucial for the virus to escape host immune recognition or attack during the early phase of the lytic life cycle of KSHV. This suggests that their promoters are primed with activating histone modifications and probably have preassembled RNA polymerase II complexes during latency as also seen with a large number of inducible cellular genes [7], [62]. However, this raises the question of how their gene expressions are suppressed during latency despite the presence of an active chromatin structure. It is intriguing that a large number of the E genes that are enriched primarily with AcH3 and H3K4me3 activating marks are also Rta-inducible, suggesting that the cooperation of Rta with the active chromatin structure may be necessary to activate expression of these E genes. Furthermore, it is conceivable that the stalled RNAPII on their promoters also requires the recruitment of specific cellular transcription factors such as PTEFb, in order to allow the conversion of RNAPII from a restricted state to an elongation-competent state [62].

Histone H3 ChIP-on-chip revealed that a small viral genomic region (between 15 and 30kb) mostly containing KSHV unique genes displayed a detectable decrease of H3 occupancy at 12hpi (Figure 1). Thus, the decrease of the repressive H3K27me3 histone mark within this region upon reactivation may potentially be a consequence of the dissociation of H3 occupancy. However, the decrease of H3 occupancy in this region does not directly correlate with the changes in histone modifications as H3K27me3 decreases in both 15–20kb and 25–30kb regions at 12 hpi, but while H3K4me3 and AcH3 increase in the 15–20kb region (ORFs 10, 11, 70, K3) they decrease in the 25–30kb region (ORFs K5, K6, K7, PAN). Thus, changes in enrichment of histone modifications seem to be gene-specific and may not necessarily be due to changes in nucleosome occupancy.

In contrast to the genome-wide repressive role of H3K27me3, the effect of the heterochromatin histone mark H3K9me3 on viral gene expression seems to be limited: enrichment of the H3K9me3 is restricted to specific genomic regions (Figure 1) and the H3K9me3 histone demethylase JMJD2A does not efficiently induce KSHV lytic replication in Vero cells (Figure 5A). However, it is possible that JMJD2A expression may induce KSHV reactivation in different cell types or H3K9me3 histone demethylases other than JMJD2A may contribute to KSHV reactivation [50]. This is in agreement with the Herpes simplex virus (HSV-1) latent genome: while the H3K9me2, H3K9me3, and H3K27me3 modifications are detected, all the tested viral promoters are most enriched in H3K27me3 [63]. On the other hand, the inhibition of the H3K9me3 histone demethylase LSD1 has been shown to block the reactivation of HSV-1 from latency [30], and the enrichment of H3K9me3 and relatively low levels of H3K27me3 were found on the latent genome of the gammaherpesvirus EBV [64], [65]. These studies indicate that H3K9me3- and H3K27me3-associated chromatin-based repression mechanisms may be a common feature of herpesvirus gene expression programs.

Genome-wide co-distribution of the EZH2 with the H3K27me3 repressive mark on lytic genes of KSHV and the EZH2 knockdown-mediated induction of lytic gene expression strongly implicate PcG proteins in the repression of lytic gene expression during latency (Figure 4, 5, S7, S10). Interestingly, our ChIP-on-chip analyses also showed that, correlating with the rise of EZH2 occupancy, H3K27me3 levels increased mostly on late gene-rich regions (30–50 kb and 105–115) during lytic reactivation. We hypothesize that in order to maintain a temporally ordered expression of lytic genes, late gene expression may be kept silenced by PcG proteins during the early gene expression period, and this repression is likely reversed only upon replication of the viral genome (Figure S1 and S2). In contrast, H3K27me3 levels significantly dropped in the 15–30kb region of the viral genome at 12 hpi, which correlates with the decrease of EZH2 binding and the dissociation of H3. Thus, decreased H3K27me3 levels may be due to the dissociation of nucleosomes, the dissociation of EZH2 and/or the recruitment of H3K27me3 histone demethylases. Nevertheless, these data suggest that the regulation of spatial and temporal association of the PcG proteins with the KSHV genome may be crucial for both KSHV latency maintenance and lytic reactivation. Indeed, SUZ12 as well as EZH2, the H3K27me3 histone methyltransferase of PcG proteins, extensively associate with the RTA promoter during latency, while EZH2 rapidly dissociates from the RTA promoter upon reactivation, which is in apparent correlation with the decrease of H3K27me3 levels and the increase of RTA expression (Figure 3A, 4B and S1). Several studies have shown that PcG proteins are recruited to their target promoters through specific DNA elements, transcription factors or non-coding RNAs, which leads to the condensation of nucleosomes, the inhibition of RNAPII elongation, and thereby repress the expression of their target genes [16], [18], [19], [20], [66], [67], [68], [69]. Several cellular and viral proteins, K-RBP, KAP-1 and LANA, are involved in the maintenance of KSHV latency, suggesting that they may be potential recruiters of PcG proteins onto the KSHV genome [70], [71], [72]. Further studies are needed to clarify how EZH2 is recruited to the KSHV genome and how EZH2 binding is modulated during reactivation. Recently, it has been also shown that BMI, a subunit of the polycomb repressive complex PRC1, is recruited onto lytic HSV-1 promoters during latency [63], [73]. Thus, the roles of PcG proteins in herpesvirus latency remain under active investigation.

Derepression of the PcG proteins-mediated silencing of gene expression have been linked to the recruitment of the H3K27me3 histone demethylases UTX and JMJD3 and the H3K4me3 histone methyltransferases MLL3 and MLL4 onto the target promoters, suggesting a tight cooperation between demethylation of the H3K27 and trimethylation of the H3K4 [23], [74]. We also found that overexpression of either UTX or JMJD3 resulted in the efficient reactivation of KSHV, suggesting that H3K27me3 histone demethylases release the PcG-mediated repression of lytic gene expressions (Figure 5A). Furthermore, our ChIP analysis (Figure 4C) indicates that along with the histone acetyltransferase CBP, the chromatin remodelling complex SWI/SNF, and the TRAP/Mediator complex [34], [38], cellular transcription factors CBF1 and RNAPII are also recruited onto the RTA promoter to induce its gene expression. Taken together, PcG proteins are deposited on the RTA promoter to suppress transcription of RTA during latency, whereas upon reactivation, the resetting of histone modifications, remodelling of the chromatin structure, and the recruitment of a large set of transcription cofactors result in the activation of RTA gene expression.

Collectively, our results indicate for the first time that histone modifications associated with the latent KSHV genome can be involved not only in the regulation of latency, but also in the control of the temporal and sequential order of the lytic gene expression cascade of KSHV upon reactivation. PcG proteins appear to play a key role in the maintenance of latency as well as in the inhibition of late gene expression during the early phase of lytic reactivation. Besides these beneficial roles of PcG proteins for the viral lifecycle, KSHV may adjust its viral genome through epigenetic modifications to tightly regulate viral expression to avoid promiscuous expression of lytic genes, which may trigger host immune system responses against KSHV infected cells.

## Materials and Methods

### Cell cultures and chemical treatments

TRExBCBL1-RTA is a KSHV-positive cell line, which expresses a doxycycline-inducible Rta gene [39]. It was maintained in RPMI 1640 medium (Cellgro) supplemented by 10% Tet system approved FBS (Clontech), 100 U/ml penicillin, 100ug/ml streptomycin and 20ug/ml hygromycin B. KSHV- and EBV-positive cell line JSC-1 was grown in RPMI 1640 medium (Cellgro) containing 10% FBS (Clontech), 100 U/ml penicillin and 100ug/ml streptomycin. For 293A, HeLa and Vero-rKSHV.219 cell lines DMEM medium (Invitrogen) containing 10% FBS (Clontech), 100 U/ml penicillin and 100ug/ml streptomycin was used. 1 ug/ml of Doxycycline (Dox) was used to induce the expression of myc/His-tagged Rta in TRExBCBL1-RTA. JSC-1 was stimulated with 5, 1, 0.2 or 0.04 uM of 3-deazaneplanocin (DZNEP) or 200 nM of staurosporine (STS).

### Plasmids, transfection and luciferase assay

Expression plasmids HA-JMJD3 and HA-UTX were obtained from Kristian Helin (University of Copenhagen). HA-JMJD2A expression vector was gift from Yang Shi (Harvard University). HA-UTXm was generated by replacing the C terminus of the UTX in HA-UTX with PCR cloning to change histidine to alanine at amino acid 1146. pLuc3kb, pLuc1.9kb and pLuc0.9kb reporter plasmids were generated by inserting the Luciferase gene derived from pGL3-Basic and different sized fragments of the promoter region of KSHV Rta into pcDNA5/FRT. 293A and Vero.219 cells were transfected by Polyfect (Qiagen) according to the manufactrer's specifications. Luciferase assay was performed by using the Promega system. The luciferase activity values are the average of at least three independent experiments.

### Immunofluorescent analysis

Vero cells were transfected with plasmids expressing HA-tagged JMJD2A, JMJD3, UTX or UTXm respectively. 3 days post transfection, cells were fixed by 4% paraformaldehyde and then permeabilized by 0.2% Triton X100. 10% goat serum was used for blocking aspecific binding of antibodies followed by incubation of cells with antibodies against HA-tag, H3K9me3 and H3K27me3. After extensive washing with PBS, FITC- and TRITC-conjugated secondary antibodies were applied followed by Hoechst staining.

### DNA affinity purification assay

Biotinylated DNA was made with biotin-conjugated primers and PCR amplification of sequences that are derived from the KSHV RTA promoter. KSHV (U75698.1 at GenBank) coordinates for fragment A and B are 69861–70500 and 70511–71130, respectively. The assay was performed essentially as described by Atanasiu et. al. [75] except that streptavidine resin (Stratagene) was used to pull down the DNA/protein complexes and proteins were eluted from the streptavidine resin with Laemmli sample buffer (Sigma).

### Antibodies for ChIPs and western blotting

The following antibodies were used in ChIPs and/or western blotting: rabbit anti-histone H3 (Abcam ab1791), rabbit anti-H3K27me3 (Millipore 07-448), rabbit anti-H3K9me3 (Millipore 07-442), rabbit anti-H3K4me3 (Millipore 04-745), rabbit anti-acetyl-histone H3 (AcH3) (Millipore 06-599), rabbit anti-RNA polymerase II (H-224) (total RNAPII) (Santa Cruz sc-9001), mouse anti-RNA polymerase II (CTD4H8) (RNAPII Ser5) (Millipore 05-623), rabbit anti-CBF1 (Abcam ab25949), mouse anti-EZH2 (BD Biosciences 612666), rabbit anti-SUZ12 (Abcam ab12073). For western blotting the following antibodies were used: mouse anti-PARP (BD Biosciences 556494), mouse anti-actin (Abcam), anti-JMJD2A (Bethyl A300-861A), anti-JMJD3 (Abgent AP1022a), KSHV specific antibodies such as mouse anti-Rta gift from Koichi Yamanishi (Osaka University, Japan), rabbit anti-K8.1, mouse anti-K8 (Abcam ab36617), rabbit anti-K2 (ABI 13-214-050), rat anti-LANA (ABI 13-210-100), Epstein Barr Virus specific antibody such as mouse anti-Zebra (Zta) (Argene 11-007).

### Chromatin Immunoprecipitation assay (ChIP)

TRExBCBL1-RTA cells (4×10^7^) were treated for 6, 12 and 24 hr with 1 ug/ml doxycycline. After fixation of the cells with 1% (v/v) formaldehyde for 10 min at RT, the cross-linking was stopped by adding glycine (final concentration 125 mM) for 5 min at RT. Cells were washed (3×) with cold PBS and then resuspended in Cell Lysis Buffer (5 mM Tris-HCl, pH 8.0, 85 mM KCl, 0.5% NP40, 1× protease inhibitor cocktail (Roche)) and incubated on ice for 10 min. After centrifugation (5 min, 5000 rpm at 4°C), the pellet was resuspended in 1.8 ml of RIPA buffer (10 mM Tris-HCl, pH 8.0, 1 mM EDTA, pH 8.0, 140 mM NaCl, 0.1% SDS, 0.1% sodium deoxycholate, 1% Triton X-100, 1 mM PMSF, 1× protease inhibitor cocktail), sonicated and centrifuged at 13000 rpm for 10 min at 4°C to remove cell debris. Aliquots of the supernatant (cellular and viral chromatin) were stored at −80°C.

For preparation of input DNA 20ul of chromatin was incubated in 100ul of TE buffer containing 50ug/ul RNase A for 30 min at 37°C. The samples were then adjusted to contain 0.5% SDS and 0.5mg/ml Proteinase K (Invitrogen) and incubated for 1 hr at 37°C. Formaldehyde crosslinks were reversed by adding sodium chloride (final concentration 300 mM) to the samples and incubated overnight at 65°C. DNA was extracted first by one volume of phenol/chloroform/isoamyl alcohol (25∶24∶1) saturated with 10 mM Tris, pH8.0 and 1 mM EDTA and then purified once by one volume of chloroform. DNA was precipitated by cold absolute ethanol, 10% (v/v) of 3 M sodium acetate, pH 5.2 and 1ul of 15 mg/ml Glycogen Blue (Ambion) at −80°C at least for 1 hr following by wash with 70% ethanol. Finally the input DNA was dried at RT and resuspended in 20ul of water.

For ChIPs chromatin containing 10ug of DNA was first diluted in 500ul of RIPA buffer and precleared by Sepharose A beads. Immunoprecipitation was carried out with 1–2ug of antibodies overnight at 4°C. Next day to pull down the DNA/protein complexes, Protein-A/G agarose was added for 4 hr. Immunoprecipitation was washed sequentially with RIPA buffer once briefly and once for 10 min followed by washing with LiCl buffer (10 mM Tris-HCl, pH 8.0, 1mM EDTA, pH 8.0, 250 mM LiCl, 0.5% NP-40, 0.5% sodium deoxycholate) once for 10 min and with TE buffer two times for 10 min. The DNA/protein-Protein A/G agarose complex was resuspended in 100ul of TE buffer containing 50ug/ul RNase A and incubated for 30 min at 37°C. The Proteinase K treatment, crosslink reversal and DNA purification was done exactly as it was described at the preparation of input DNA. Both input and ChIP DNAs were measured by qPCR. Based on the standard curves for each primer pairs the enrichment of proteins and histone modifications on specific genomic regions were calculated as percentage of the immunoprecipitated DNA compared to input DNA. Each data points in ChIP figures were averages of at least three independent ChIPs using three independent chromatins.

Sequential ChIP (seqChIP) was performed as follows. ChIPs were carried out as described above except that after the first ChIP (ChIP I.) the immunoprecipitated DNA/protein complexes were eluted from the Protein A/G agarose by 100ul of Elution buffer (50 mM Tris-HCl, pH 7.5, 10 mM EDTA, pH 8.0, 1% SDS) by heating for 10–15 min at 65°C. 10% of the elution was saved for qPCR while the rest was diluted with RIPA buffer up to 1 ml and the second immunoprecipitation (ChIP II.) was performed exactly as described above for ChIP. The second ChIP was also measured by qPCR and the enrichment of the histone modifications on specific genomic regions was calculated as percentage of the immunoprecipitated DNA compared to the total amount of DNA eluted after the first ChIP. Each ChIP values were averages of three independent ChIPs using three independent chromatins.

### Quantitative real-time PCR (qPCR)

qPCR was performed using iQ SYBR Green Supermix (Bio-Rad) and CFX96 real-time PCR machine (Bio-Rad). PCR program was as follows: after an initial preincubation step at 95°C for 3 min, there were 40 cycles, each consisting of 95°C for 10sec, 64.5 or 59°C depending on the primers (Table S1) for 20 sec and 72°C for 20sec. The last amplification cycle was followed by a melt curve analysis to make sure about the specificity of the qPCR amplification. As for ChIP 0.45ul of ChIP DNA and 4.5ng of input DNA were measured in qPCR. Quantification of the amplifications in qPCR was based on standard curves for each primer pairs. Sequences of the primers used in ChIP experiments can be found in Table S1.

### RNA isolation and RT-PCR

Total cellular RNA was extracted with Tri reagent (Sigma) according to the manufacturer's instructions. 1ug of total RNA was treated by DNase I (Sigma), reverse transcribed by iScript cDNA Synthesis kit (Bio-Rad) and the cDNA was measured by either conventional PCR or qPCR. The relative quantification of gene expression was calculated with the ddCt method, where actin mRNA was used for normalization. RT-PCR graphs were made based on the average of at least two independent experiments.

### Lentiviral shRNA knockdown

The shRNA constructs were prepared by using the pLKO.1 lentiviral vector. The target sequences are listed in Table S1. Supernatants from 293T cells transfected by the shRNA and packaging vectors were collected 60 hours post transfection followed by concentration of the virus (24000 rpm, 1.5 hr, 4°C) and used for spinning infections (1800 rpm, 45 min) of one million of BCBL-1 cells in the presence of 10 ug/ml polybrene. 5 days post infection cells were harvested for western blot and RT-qPCR analysis.

### KSHV tiling microarray and ChIP-on-chip

The 15 bp-tiling KSHV microarray contains both KSHV and human probes and was manufactured by Agilent Technologies. The probes were spotted on 8×15K array format. The majority of the oligonucleotides are overlapping 60-mer probes covering the entire KSHV genome (U75698 and U75699 at GenBank). In addition, probes specific for the gene regulatory regions of 71 human genes were also included in the microarray. The human probes are derived from the Agilent Human CoC 2×244k microarray design. For ChIP-on-chip 30 ug of chromatin and 5–6 ug of antibodies were used per ChIP. The ChIP and DNA purification was performed exactly as described above. ChIP DNA was amplified by Complete Whole Genome Amplification (WGA2) kit (Sigma-Aldrich) and purified with QIAquick PCR Purification kit (Qiagen) according to the instructions of the manufacturers. Labeling, hybridisation and scanning of the microarrays were done at the Functional Genomics Core, Microarray Service at City of Hope (California). Probe signals were extracted using the Agilent Feature Extraction software (version 10.5.1.1). Each ChIP-on-chip experiment was performed two times and the average of the biological replicates is shown in Figures 1, 2, 4A, S5, S6, S8. The biological replicates can be found in Figures S3, S4, S5.

### Normalization, visualization and cluster analysis of the ChIP-on-chip data

Log_2_ (Cy5/Cy3) of the mean signals of the probes were calculated, and imported into R software (version 2.9.0) (http://www.r-project.org). Using the Bioconductor package “aroma.light,” lowess normalization as implemented by the function “normalizeLoess,” was used to correct for die bias. The log_2_ ratios for samples at 0hpi and 12hpi were scaled to have the same median absolute deviation (MAD) using the function “normalizeBetweenArrays,” from the “limma” Bioconductor package [76], [77], [78], [79].

To visualize enrichment across the KSHV genome, the normalized log_2_ ratios for each overlapping probe were averaged and a score was assigned to each base pair (bp). A moving average was calculated across the genome by sliding a 150-bp window stepwise by 1 bp unidirectionally. When calculating the moving average, the window is shifted past regions where there are missing probes so that regions with missing probes do not have any weight. Regions where no probes exist were assigned an enrichment value of “0.” Log_2_ ratios were converted to linear scale, and tracks in BED format were created for each sample. Tracks were then imported into Genetrix software (Epicenter Software) for visualization.

For the KSHV promoter array analysis promoter regions were defined as 1000 bp upstream and downstream of the translational start sites (TSS) of each viral gene. The coordinates of ORFs are listed in Table S2 [80]. Normalized log_2_ ratios were calculated as described above. Log_2_ ratios were averaged for each 50 bp nonoverlapping window upstream and downstream of the TSS within the promoter for each gene. The TSS was assigned a single score. The resulting matrix was applied to perform hierarchical clustering with Cluster 3.0 (http://bonsai.ims.u-tokyo.ac.jp/~mdehoon/software/cluster/software.htm). The results of clustering were then imported into Java TreeView for visualization (version 1.1.4r2) [81].

While the 0 hpi-ChIPs were compared to the 0 hpi-DNA inputs in ChIP-on-chips the 12 hpi-ChIP-on-chips were analysed in two different ways. (1) 12 hpi-ChIPs were compared to 0 hpi-ChIPs (Figure S3, S4, S5, S6, S7, S8, S10) or (2) the 12 hpi-ChIP/input ratio was calculated by multiplying the 0 hpi-ChIP/input ratios by the 12 hpi-ChIP/0 hpi-ChIP ratios (Figure 1 and 2).

## Supporting Information

Figure S1 RTA-mediated reactivation of KSHV. **(A)** TRExBCBL1-RTA cells were treated by 1 ug/ml of doxycycline for 6, 12 and 24 hours and subject to immunoblot analysis with the indicated antibodies. myc/His-Rta was detected by anti-myc antibody. Actin was used to monitor protein amounts in the cell lysates. **(B)** An aliquot of cells used in (A) was used for purification of total RNA to measure the indicated viral mRNAs. **(C)** Using the same number of cells, the copy number of KSHV genome in Dox-induced (6, 12, 24 hpi) TRExBCBL1-RTA cells relative to that in non-induced (0 hpi) cells were determined by qPCR using primers specific for the indicated genomic regions. The amount of viral DNA was normalized for the cellular DNA input.
(0.35 MB TIF)

Figure S2 Dissociation of histone H3 from the replicating KSHV genome. **(A)** The schematic map shows the KSHV genome and arrows indicate the KSHV genomic regions tested in ChIPs. RTA and LANA promoters are shown in more details. 1 to 16 represents genomic regions spanning the RTA promoter and the intron of RTA (+0.8 kb) relative to the translational start site of RTA. CBF1-binding sites are highlighted in blue. **(B)** Non-induced and Dox-induced TRExBCBL1-RTA cells were used to perform ChIPs for hisone H3 on different viral gene promoters or **(C)** on cellular promoters. **(D)** Non-induced and Dox-induced TRExBCBL1-RTA cells were tested in immunoblot for histone H3 and indicated histone modifications.
(0.59 MB TIF)

Figure S3 Genome-wide mapping of histone modifications on the KSHV genome during latency and reactivation. It shows one of the biological replicates of the ChIP-on-chip experiments (Chromatin A). Details are described in Figure 1 and S7. (**A**) ChIP-on-chip for histone H3. (**B**) Histone modifications on the KSHV genome during latency. (**C**) Changes of histone modification on the KSHV genome upon lytic reactivation.
(0.88 MB TIF)

Figure S4 Genome-wide mapping of histone modifications on the KSHV genome during latency and reactivation. It shows one of the biological replicates of the ChIP-on-chip experiments (Chromatin B). Details are described in Figure 1 and S7. (**A**) ChIP-on-chip for histone H3. (**B**) Histone modifications on the KSHV genome during latency. (**C**) Changes of histone modification on the KSHV genome upon lytic reactivation.
(0.88 MB TIF)

Figure S5 Normalization of the ChIP-on-chip data by histone H3 at 0 hpi. The ChIP-on-chip experiments were performed as described in Figure 1. The 0 hpi-histone modification ChIP-on-chip data were derived from two independent chromatins (Chromatin A and B), which were divided by the relevant 0 hpi-H3 ChIP-on-chip dataset (the upper and middle panels). The average of the H3-normalized 0 hpi-ChIP-on-chip dataset is shown in the lower panel.
(0.67 MB TIF)

Figure S6 Comparison of the hierarchical clustering of histone modifications associated with the regulatory regions of viral genes with and without H3 normalization. The clustering was performed as described in Figure 2. (**A**) The 0 hpi-ChIP-on-chip dataset used in the hierarchical clustering without normalization for histone H3. The clustering was taken from Figure 2A. (**B**) The 0 hpi-ChIP-on-chip dataset used in the hierarchical clustering was normalized for changes in histone H3.
(2.96 MB TIF)

Figure S7 Genome-wide mapping of histone modifications on the KSHV genome during latency and reactivation. Each ChIP-on-chip experiment is an average of two biological replicates. (**A**) Histone H3 ChIP-on-chip is the same as in Figure 1. (**B**) Histone modification ChIP-on-chips were performed using non-induced TRExBCBL1-Rta cells. (**C**) ChIP-on-chips were performed with TRExBCBL1-Rta cells induced by doxycycline for 12 hours. Changes of the distribution of histone modifications during reactivation (12 hpi) are shown as the normalized Cy5/Cy3 ratio of 12 hpi-ChIP DNA over 0 hpi-ChIP DNA. Red line indicates that the Cy5 (ChIP at 12 hpi)/Cy3 (ChIP at 0 hpi) ratio equals one showing that there is no change in the level of histone modifications between 0hpi and 12hpi. Numbers in the left upper corners show the maximum values of Cy5/Cy3. Missing probes in specific genomic regions are shown below the genome scale (**). The alternating dark and light blue squares atop display the viral ORFs where the white triangle indicates ORFs that are expressed from the reverse DNA strand. The hpi stands for hours post-induction.
(0.85 MB TIF)

Figure S8 Hierarchical clustering of histone modifications associated with the regulatory regions of viral genes. Based on their expression patterns the viral genes were grouped as latent, IE, E and L genes and hierarchical clustering was performed within the groups. Details are described in Figure 2. Panel A is identical with Figure 2A while panel B shows 12 hpi-ChIP/0 hpi-ChIP based on Figure S7.
(2.64 MB TIF)

Figure S9 IE genes are repressed during latency but rapidly induced upon reactivation. Total RNAs were purified from non-induced (0 hpi) and induced (6, 12, 24 hpi) TRExBCBL1-RTA cells followed by RT-PCR using specific primers for the indicated IE transcripts. RT+: cDNA synthesis was performed with reverse transcriptase, RT−: cDNA synthesis reaction did not include reverse transcriptase.
(0.25 MB TIF)

Figure S10 Colocalization of EZH2 and H3K27me3 on the regulatory region of KSHV genes. Hierarchical clustering of EZH2 and H3K27me3 associated with the regulatory region of KSHV genes is performed as described in Figure 2.
(1.34 MB TIF)

Figure S11 Binding of CBF1 to the RTA promoter is essential for RTA-mediated activation of its own promoter. **(A)** Schematic diagrams of deletion mutants of the RTA promoter fused to the luciferase reporter gene. The size of the tested promoter regions relative to the translational start site of RTA is indicated on the left. **(B)** The deletion mutants were transfected into 293A cells in the absence or presence of RTA and assayed for luciferase activity. Data represent the average of three independent experiments. **(C)** Nuclear extract of BCBL1 was subject to DNA affinity purification with DNA fragments derived from the RTA promoter region. Fragment A includes the 3 CBF1 binding sites at −1.4 kb relative to the translational start site of RTA. Fragment B is derived from the adjacent promoter region of Fragment A that does not contain any CBF1 binding sites. Immunoblot was performed with an anti-CBF-1 specific antibody.
(0.13 MB TIF)

Figure S12 Overexpression of histone demethylases in Vero cells. **(A)** Overexpressed HMTs were detected by immunoblotting with HA-, JMJD2A- and JMJD3-specific antibodies. **(B and C)** HA-tagged JMJD2A, JMJD3, UTX and UTXm were transfected into Vero cells, which were subject to immunofluorescent analysis at 3 days post-transfection. The expression of enzymatically active JMJD2A suppressed the steady-state level of H3K9me3, whereas JMJD3 and UTX decreased H3K27me3 in transfected cells.
(1.27 MB TIF)

Figure S13 Induction of apoptosis is not sufficient to induce KSHV reactivation. **(A)** JSC-1 cells were treated with 5 uM DZNep for 1, 2 and 3 days (dpt) and then harvested for immunoblot analysis with an anti-PARP antibody. **(B)** JSC-1 cells were treated with different concentrations of DZNep for 3 days and then subject to immunoblot analysis with the indicated antibodies. **(C)** JSC-1 cells were treated with 200 nM of staurosporine (STS) for 1, 2 and 3 days followed by immunoblot analysis for the indicated cellular proteins and histone modifications (H3K27me3, H3K9me3). **(D)** A portion of control and 3dpt JSC-1cells used in (C) was used for RNA purification and the levels of the indicated KSHV mRNA were measured by RT-qPCR.
(0.55 MB TIF)

Table S1 Primer sequences
(0.06 MB DOC)

Table S2 Genomic coordinates of the open reading frames of KSHV. IE = immediate early, E = early, L = late. Start codon coordinates indicate the first nucleotide of the KSHV genes and the stop codon coordinates indicate the last nucleotide in the stop codon of the KSHV genes. Coordinates are based on U75698.1 at GenBank.
(0.05 MB DOC)
