# Supplementary figures and images for: Epigenetic Analysis of KSHV Latent and Lytic Genomes
Source: PLoS Pathog. 2010 Jul 22;6(7):e1001013. doi: 10.1371/journal.ppat.1001013 (PMC2908616; doi:10.1371/journal.ppat.1001013)

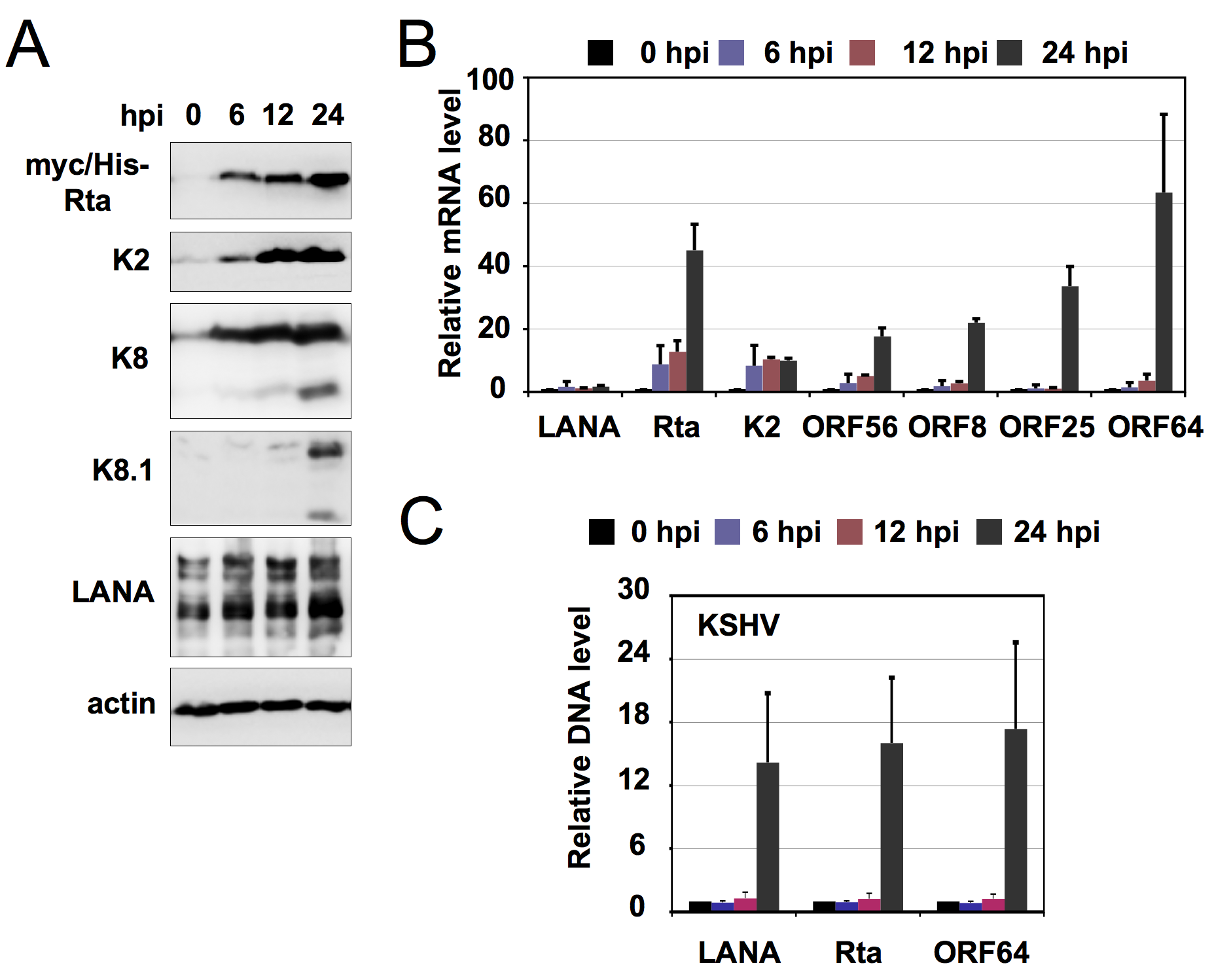

Supplement: Figure S1 — RTA-mediated reactivation of KSHV. (A) TRExBCBL1-RTA cells were treated by 1 ug/ml of doxycycline for 6, 12 and 24 hours and subject to immunoblot analysis with the indicated antibodies. myc/His-Rta was detected by anti-myc antibody. Actin was used to monitor protein amounts in the cell lysates. (B) An aliquot of cells used in (A) was used for purification of total RNA to measure the indicated viral mRNAs. (C) Using the same number of cells, the copy number of KSHV genome in Dox-induced (6, 12, 24 hpi) TRExBCBL1-RTA cells relative to that in non-induced (0 hpi) cells were determined by qPCR using primers specific for the indicated genomic regions. The amount of viral DNA was normalized for the cellular DNA input. (0.35 MB TIF) [file ppat.1001013.s001.tif]

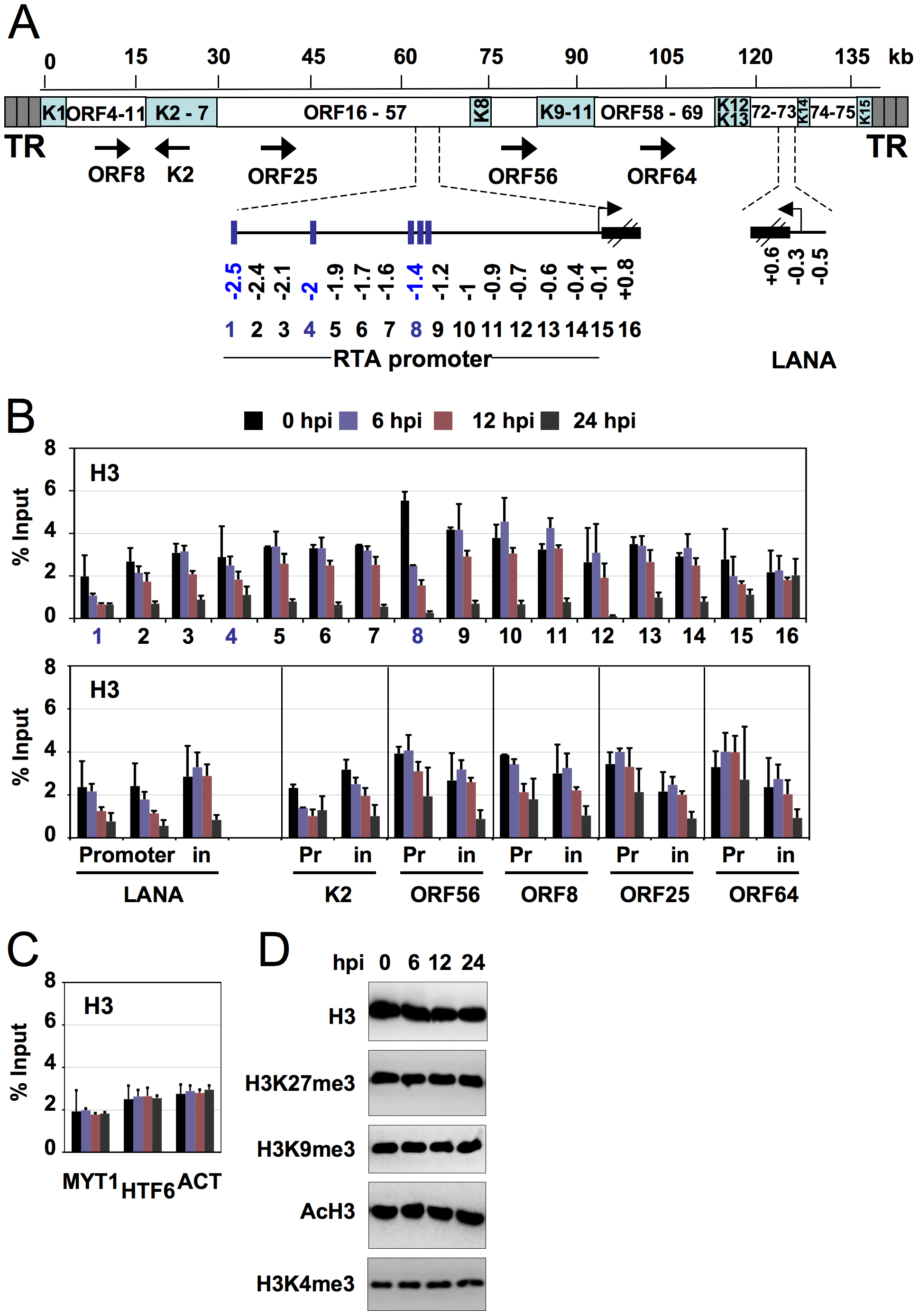

Supplement: Figure S2 — Dissociation of histone H3 from the replicating KSHV genome. (A) The schematic map shows the KSHV genome and arrows indicate the KSHV genomic regions tested in ChIPs. RTA and LANA promoters are shown in more details. 1 to 16 represents genomic regions spanning the RTA promoter and the intron of RTA (+0.8 kb) relative to the translational start site of RTA. CBF1-binding sites are highlighted in blue. (B) Non-induced and Dox-induced TRExBCBL1-RTA cells were used to perform ChIPs for hisone H3 on different viral gene promoters or (C) on cellular promoters. (D) Non-induced and Dox-induced TRExBCBL1-RTA cells were tested in immunoblot for histone H3 and indicated histone modifications. (0.59 MB TIF) [file ppat.1001013.s002.tif]

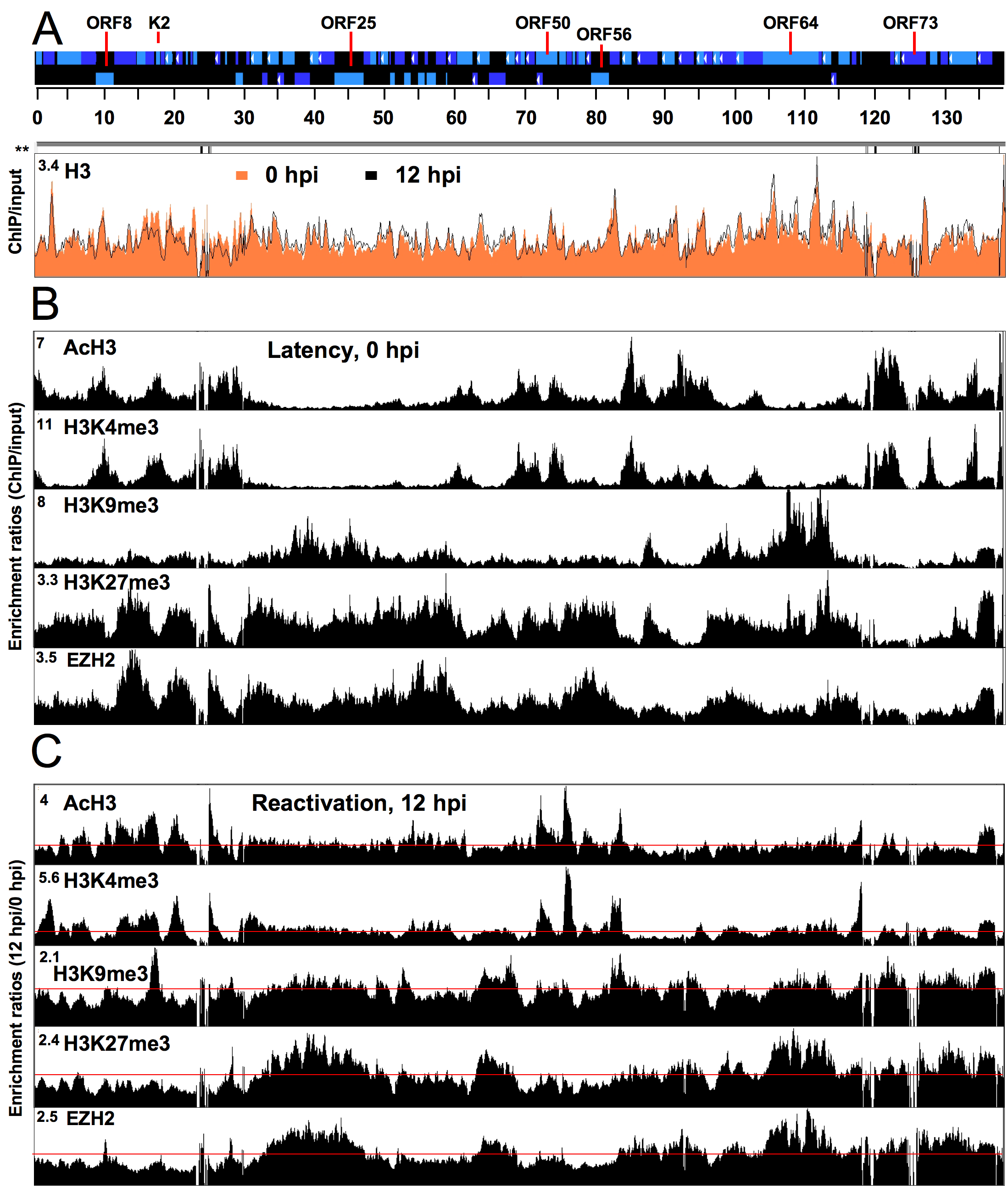

Supplement: Figure S3 — Genome-wide mapping of histone modifications on the KSHV genome during latency and reactivation. It shows one of the biological replicates of the ChIP-on-chip experiments (Chromatin A). Details are described in Figure 1 and S7. (A) ChIP-on-chip for histone H3. (B) Histone modifications on the KSHV genome during latency. (C) Changes of histone modification on the KSHV genome upon lytic reactivation. (0.88 MB TIF) [file ppat.1001013.s003.tif]

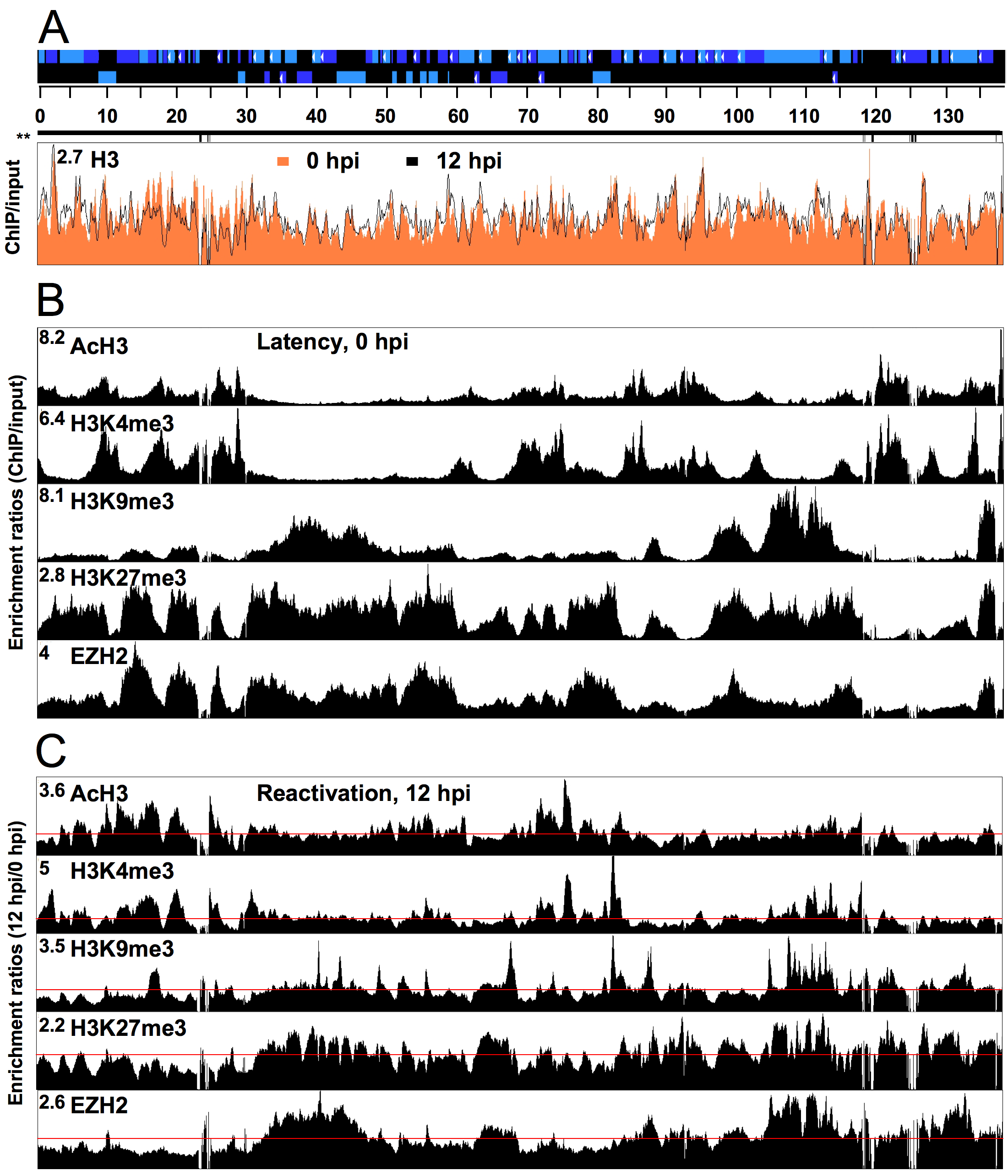

Supplement: Figure S4 — Genome-wide mapping of histone modifications on the KSHV genome during latency and reactivation. It shows one of the biological replicates of the ChIP-on-chip experiments (Chromatin B). Details are described in Figure 1 and S7. (A) ChIP-on-chip for histone H3. (B) Histone modifications on the KSHV genome during latency. (C) Changes of histone modification on the KSHV genome upon lytic reactivation. (0.88 MB TIF) [file ppat.1001013.s004.tif]

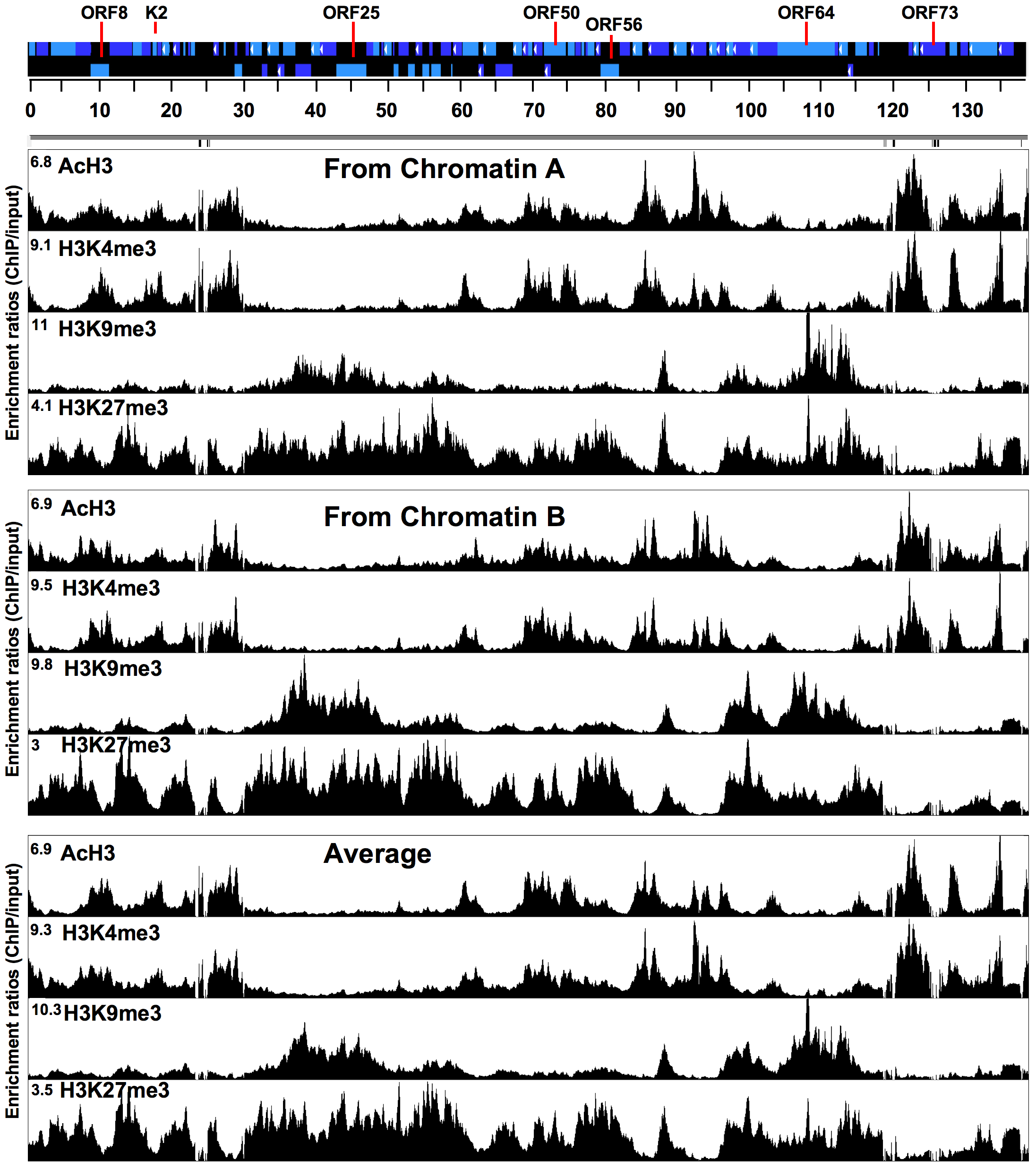

Supplement: Figure S5 — Normalization of the ChIP-on-chip data by histone H3 at 0 hpi. The ChIP-on-chip experiments were performed as described in Figure 1. The 0 hpi-histone modification ChIP-on-chip data were derived from two independent chromatins (Chromatin A and B), which were divided by the relevant 0 hpi-H3 ChIP-on-chip dataset (the upper and middle panels). The average of the H3-normalized 0 hpi-ChIP-on-chip dataset is shown in the lower panel. (0.67 MB TIF) [file ppat.1001013.s005.tif]

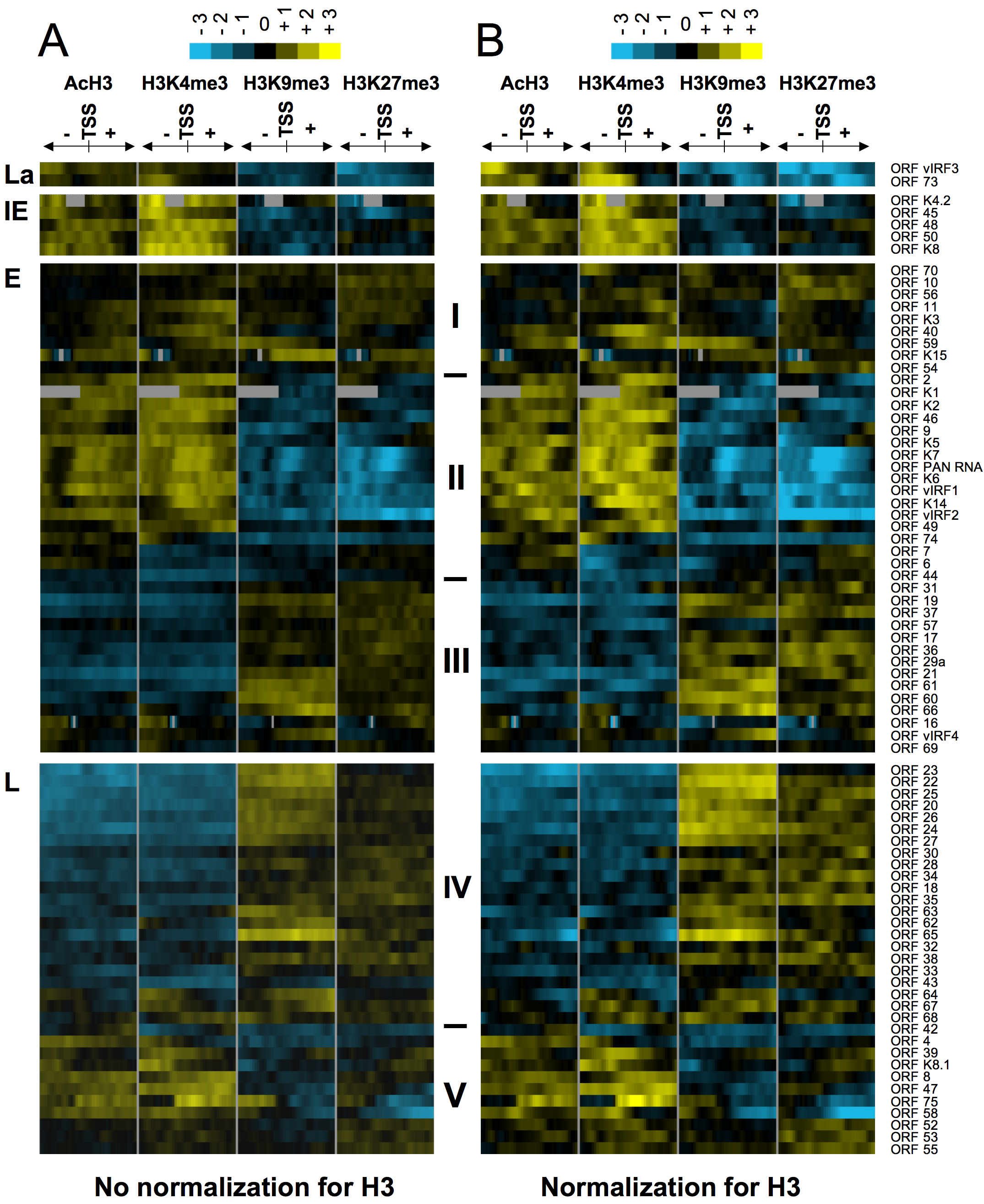

Supplement: Figure S6 — Comparison of the hierarchical clustering of histone modifications associated with the regulatory regions of viral genes with and without H3 normalization. The clustering was performed as described in Figure 2. (A) The 0 hpi-ChIP-on-chip dataset used in the hierarchical clustering without normalization for histone H3. The clustering was taken from Figure 2A. (B) The 0 hpi-ChIP-on-chip dataset used in the hierarchical clustering was normalized for changes in histone H3. (2.96 MB TIF) [file ppat.1001013.s006.tif]

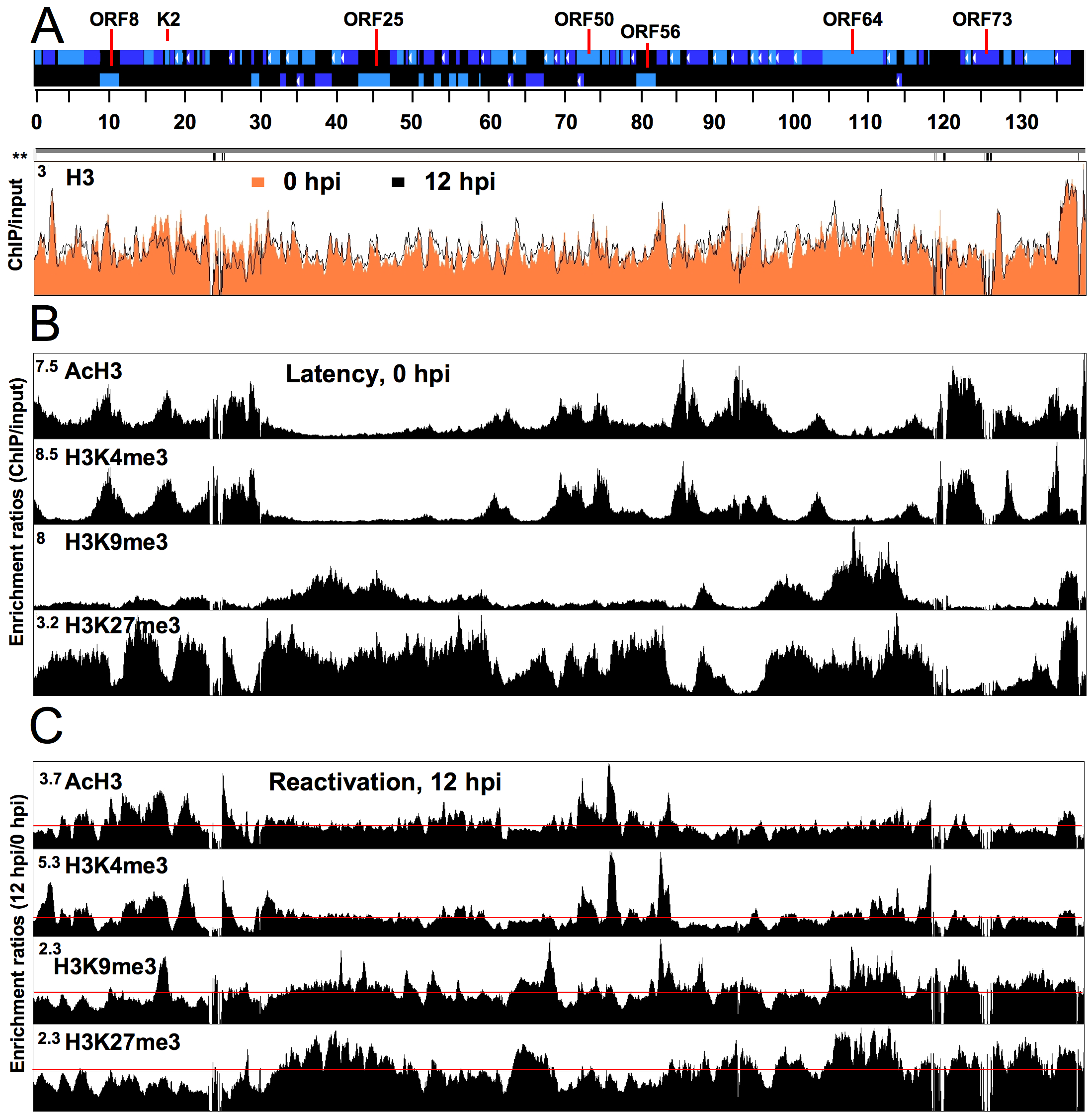

Supplement: Figure S7 — Genome-wide mapping of histone modifications on the KSHV genome during latency and reactivation. Each ChIP-on-chip experiment is an average of two biological replicates. (A) Histone H3 ChIP-on-chip is the same as in Figure 1. (B) Histone modification ChIP-on-chips were performed using non-induced TRExBCBL1-Rta cells. (C) ChIP-on-chips were performed with TRExBCBL1-Rta cells induced by doxycycline for 12 hours. Changes of the distribution of histone modifications during reactivation (12 hpi) are shown as the normalized Cy5/Cy3 ratio of 12 hpi-ChIP DNA over 0 hpi-ChIP DNA. Red line indicates that the Cy5 (ChIP at 12 hpi)/Cy3 (ChIP at 0 hpi) ratio equals one showing that there is no change in the level of histone modifications between 0hpi and 12hpi. Numbers in the left upper corners show the maximum values of Cy5/Cy3. Missing probes in specific genomic regions are shown below the genome scale (**). The alternating dark and light blue squares atop display the viral ORFs where the white triangle indicates ORFs that are expressed from the reverse DNA strand. The hpi stands for hours post-induction. (0.85 MB TIF) [file ppat.1001013.s007.tif]

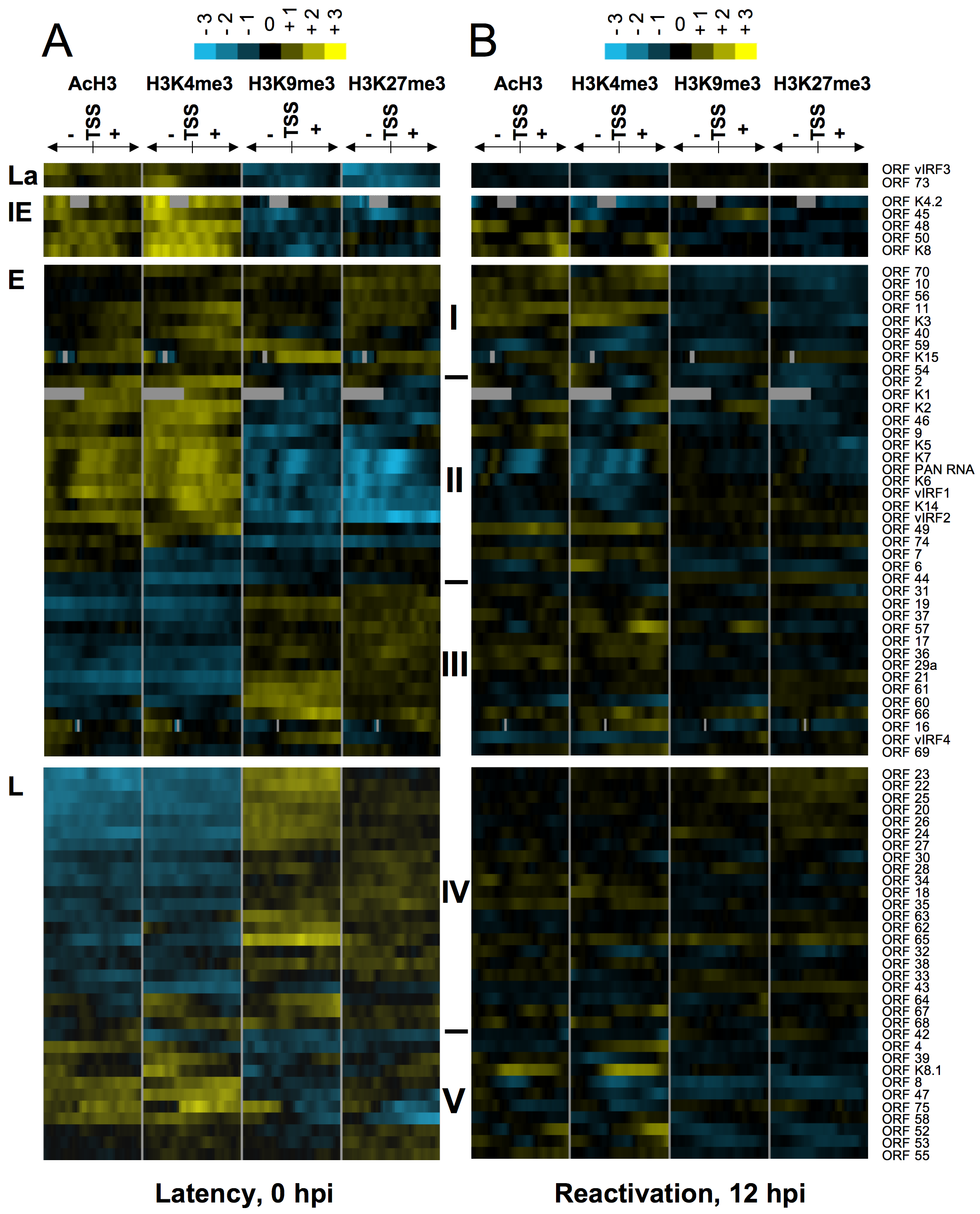

Supplement: Figure S8 — Hierarchical clustering of histone modifications associated with the regulatory regions of viral genes. Based on their expression patterns the viral genes were grouped as latent, IE, E and L genes and hierarchical clustering was performed within the groups. Details are described in Figure 2. Panel A is identical with Figure 2A while panel B shows 12 hpi-ChIP/0 hpi-ChIP based on Figure S7. (2.64 MB TIF) [file ppat.1001013.s008.tif]

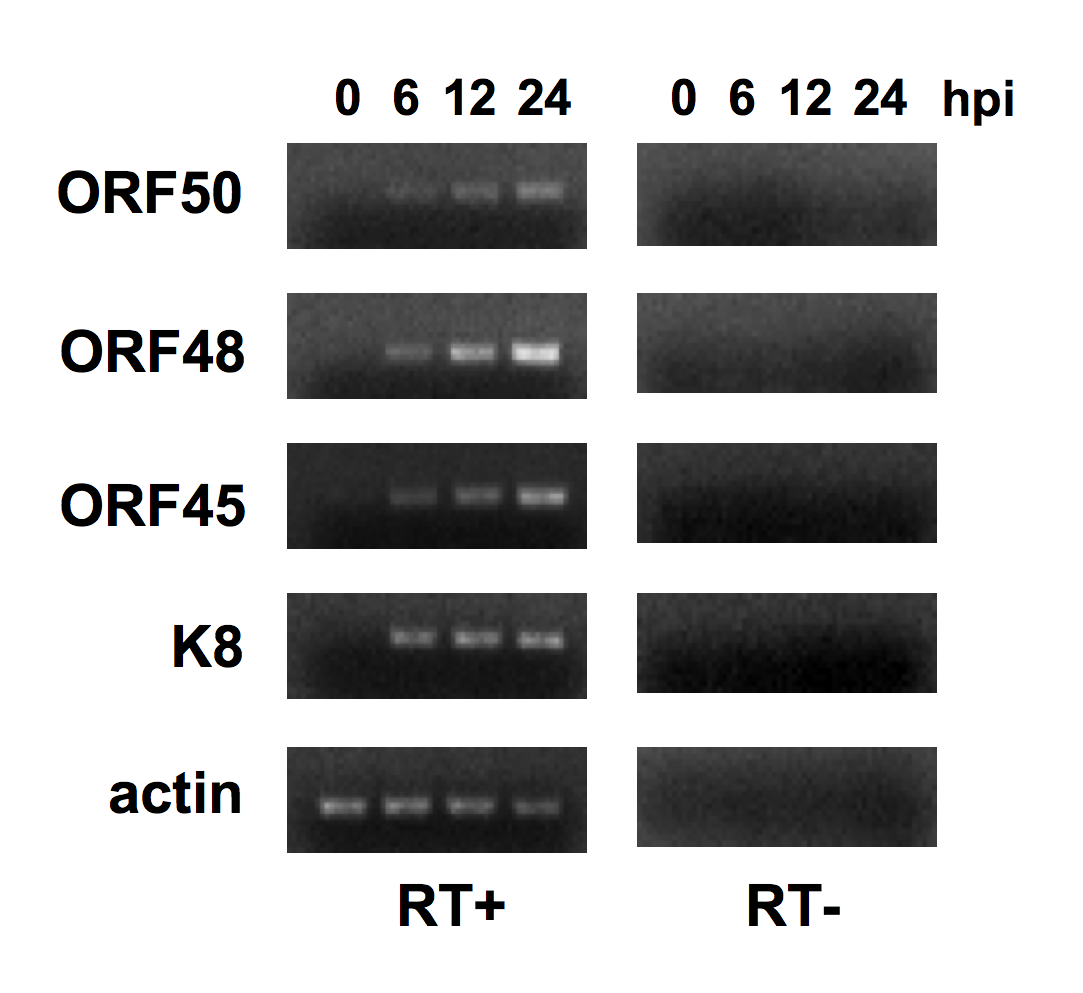

Supplement: Figure S9 — IE genes are repressed during latency but rapidly induced upon reactivation. Total RNAs were purified from non-induced (0 hpi) and induced (6, 12, 24 hpi) TRExBCBL1-RTA cells followed by RT-PCR using specific primers for the indicated IE transcripts. RT+: cDNA synthesis was performed with reverse transcriptase, RT−: cDNA synthesis reaction did not include reverse transcriptase. (0.25 MB TIF) [file ppat.1001013.s009.tif]

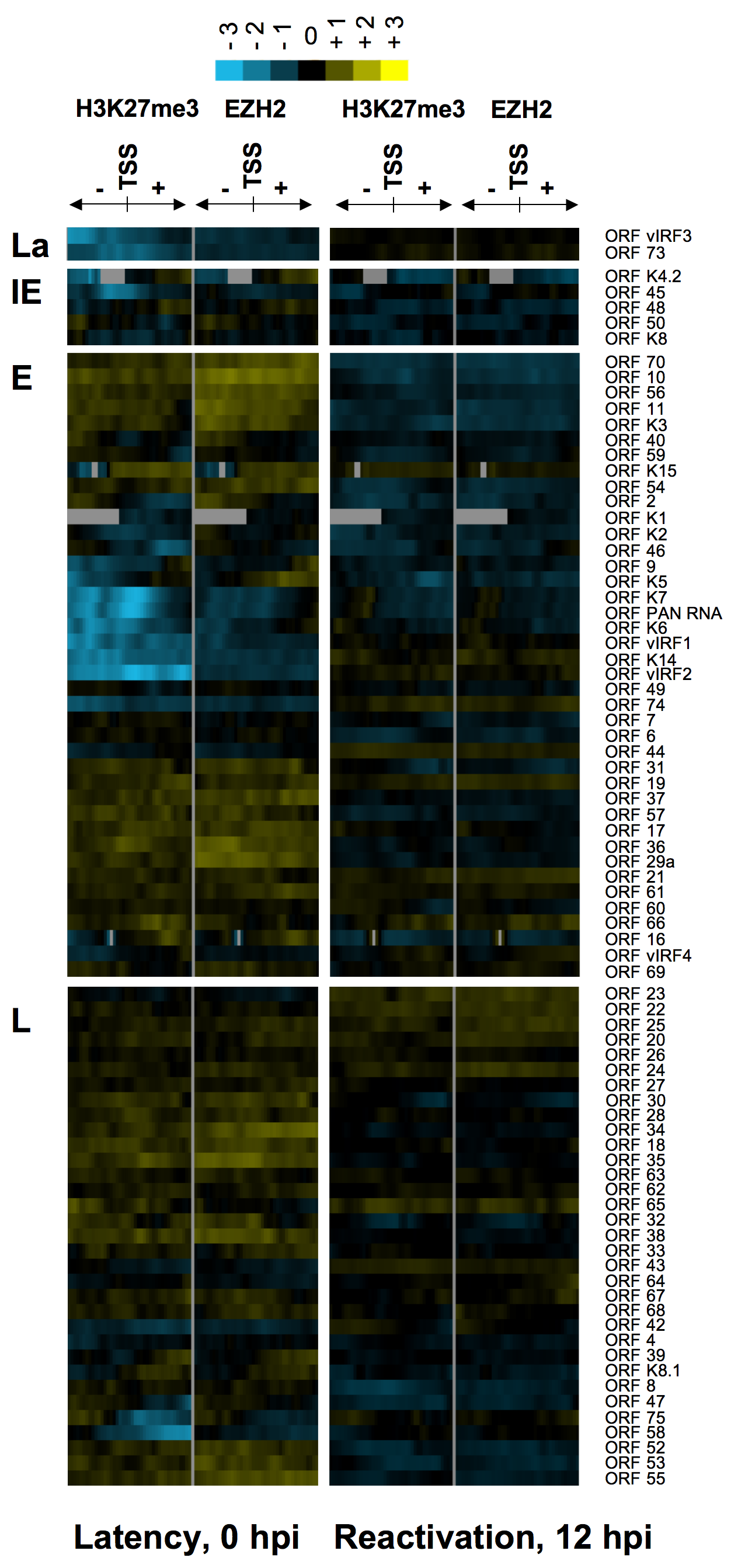

Supplement: Figure S10 — Colocalization of EZH2 and H3K27me3 on the regulatory region of KSHV genes. Hierarchical clustering of EZH2 and H3K27me3 associated with the regulatory region of KSHV genes is performed as described in Figure 2. (1.34 MB TIF) [file ppat.1001013.s010.tif]

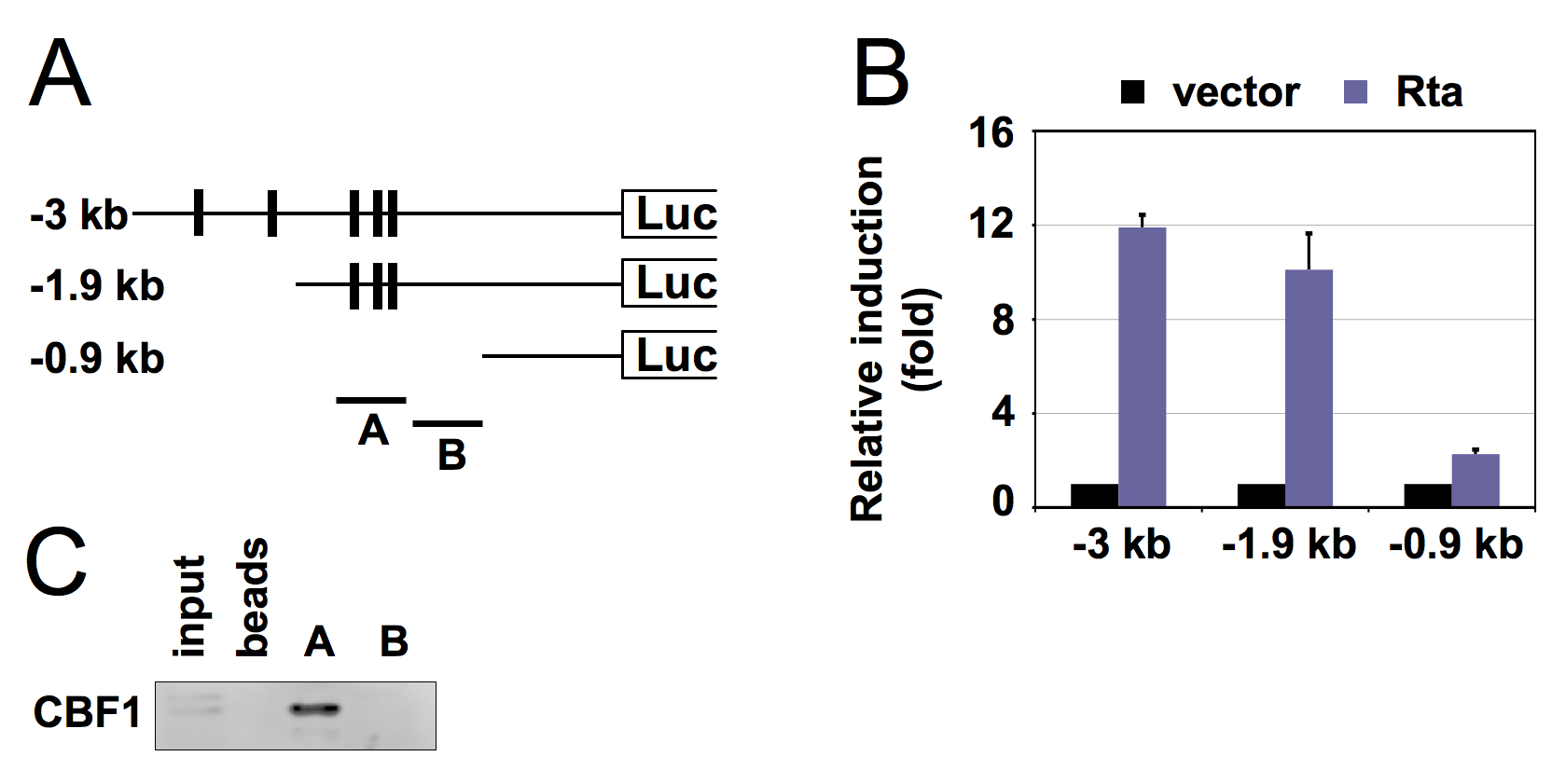

Supplement: Figure S11 — Binding of CBF1 to the RTA promoter is essential for RTA-mediated activation of its own promoter. (A) Schematic diagrams of deletion mutants of the RTA promoter fused to the luciferase reporter gene. The size of the tested promoter regions relative to the translational start site of RTA is indicated on the left. (B) The deletion mutants were transfected into 293A cells in the absence or presence of RTA and assayed for luciferase activity. Data represent the average of three independent experiments. (C) Nuclear extract of BCBL1 was subject to DNA affinity purification with DNA fragments derived from the RTA promoter region. Fragment A includes the 3 CBF1 binding sites at −1.4 kb relative to the translational start site of RTA. Fragment B is derived from the adjacent promoter region of Fragment A that does not contain any CBF1 binding sites. Immunoblot was performed with an anti-CBF-1 specific antibody. (0.13 MB TIF) [file ppat.1001013.s011.tif]

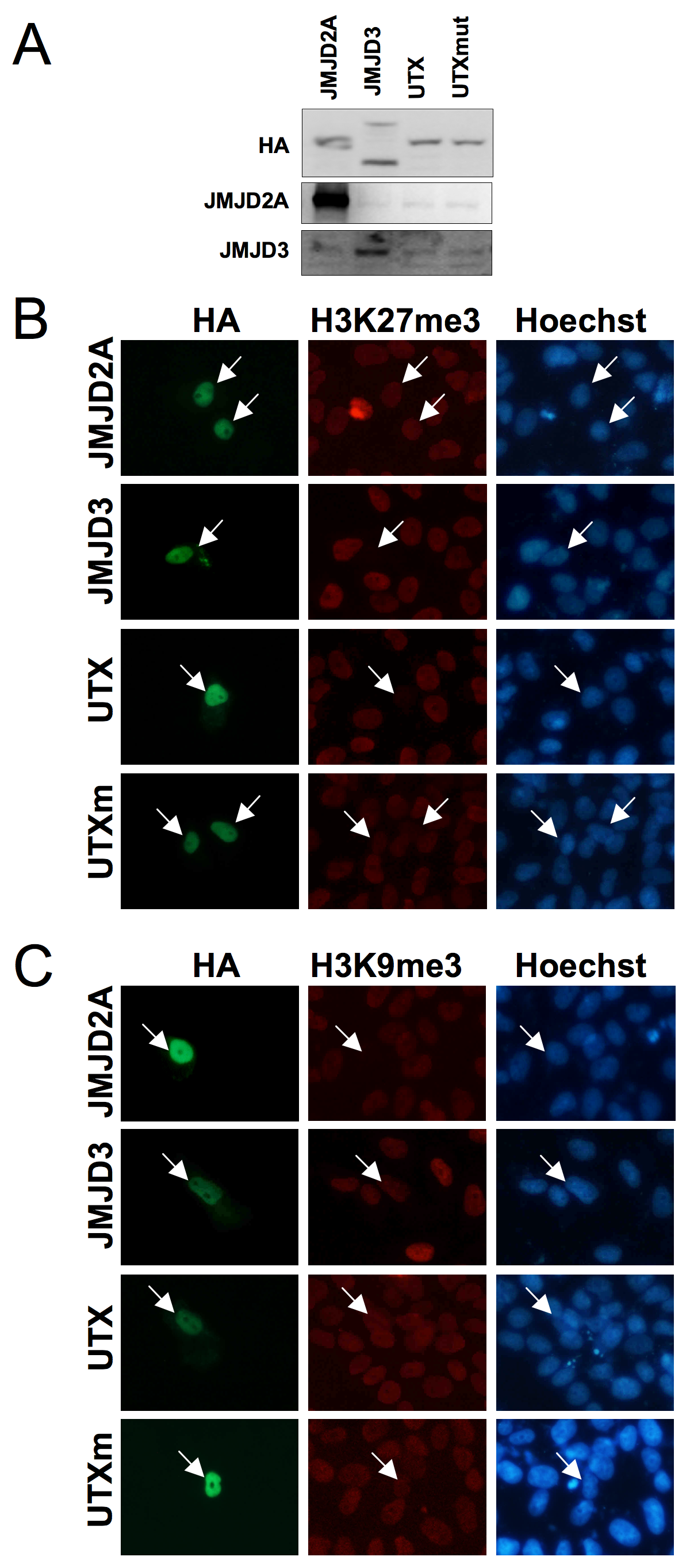

Supplement: Figure S12 — Overexpression of histone demethylases in Vero cells. (A) Overexpressed HMTs were detected by immunoblotting with HA-, JMJD2A- and JMJD3-specific antibodies. (B and C) HA-tagged JMJD2A, JMJD3, UTX and UTXm were transfected into Vero cells, which were subject to immunofluorescent analysis at 3 days post-transfection. The expression of enzymatically active JMJD2A suppressed the steady-state level of H3K9me3, whereas JMJD3 and UTX decreased H3K27me3 in transfected cells. (1.27 MB TIF) [file ppat.1001013.s012.tif]

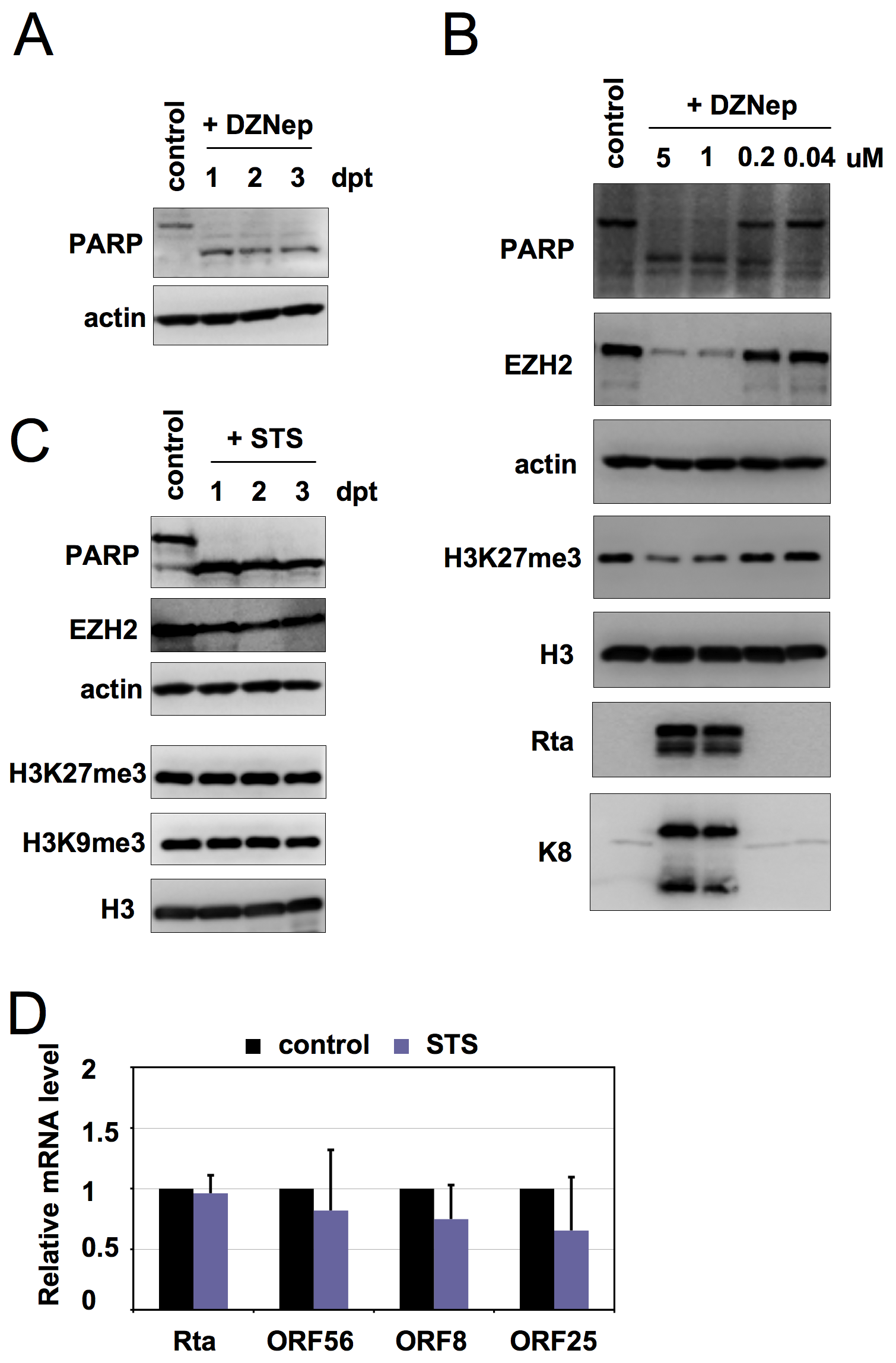

Supplement: Figure S13 — Induction of apoptosis is not sufficient to induce KSHV reactivation. (A) JSC-1 cells were treated with 5 uM DZNep for 1, 2 and 3 days (dpt) and then harvested for immunoblot analysis with an anti-PARP antibody. (B) JSC-1 cells were treated with different concentrations of DZNep for 3 days and then subject to immunoblot analysis with the indicated antibodies. (C) JSC-1 cells were treated with 200 nM of staurosporine (STS) for 1, 2 and 3 days followed by immunoblot analysis for the indicated cellular proteins and histone modifications (H3K27me3, H3K9me3). (D) A portion of control and 3dpt JSC-1cells used in (C) was used for RNA purification and the levels of the indicated KSHV mRNA were measured by RT-qPCR. (0.55 MB TIF) [file ppat.1001013.s013.tif]
