# Supplementary material for: Epigenetic Analysis of KSHV Latent and Lytic Genomes
Source: PLoS Pathog. 2010 Jul 22;6(7):e1001013. doi: 10.1371/journal.ppat.1001013 (PMC2908616; doi:10.1371/journal.ppat.1001013)
Supplement: Table S1 — Primer sequences (0.06 MB DOC) [file ppat.1001013.s014.doc]

**Table S1.** Primer sequence information

**Primer for Primer sequence 5'-3' Temp. (^o^C) Assay**

**RTA region**

- 2.5 kb Fw TCTCCACCGTCAAAACTGTGT 64.5 ChIP

Rev CGTCCCCAATTTCAGTACTCC

-2.4 kb Fw AAACTGGGTGGAACCTGACAT 64.5 ChIP

Rev GCTGCGATCCAGAGGATATTA

-2.1 kb Fw TCCACTTAGGTGCCAGTAACA 64.5 ChIP

Rev GGCTAAAAGCAGCAGCCGGAG

-2 kb Fw CTGACTTCCGCTAACAGAACCT 64.5 ChIP

Rev TGCAGATAGCTCTATTCACAACG

-1.9 kb Fw TATAGGCCGTCAAACTTCGTT 64.5 ChIP

Rev GCGTGAATGGGTTTAATCTGA

-1.7 kb Fw GATCGGCGAAGTGGATAGAGT 64.5 ChIP

Rev CCCTATTGGTCACATCTCACG

-1.6 kb Fw TCCACAGGACGGCAAATAG 64.5 ChIP

Rev TCCTCATTAGTCGGGACTCG

-1.4 kb Fw TGAGGTCTATTTCCCACGACA 64.5 ChIP

Rev ACAGCTCCGACGATGAGTATG

-1.2 kb Fw CATCGGAATCATCAGCACAC 64.5 ChIP

Rev TGGACATTTCAAACCCATCA

-1 kb Fw CCCCAACACAAGGACCTTTA 64.5 ChIP

Rev GCTTTTGGATACCCTGGTGA

-0.9 kb Fw CGGCTTACCGACAGTTCTTT 64.5 ChIP

Rev GAGTATGGCGGACTGTCACC

-0.7 kb Fw TTCAAACACGATGAAATCAAGC 64.5 ChIP

Rev ATACAGACACCGGAGCAATACC

-0.6 kb Fw AAGACACTGACCCACCAAGG 64.5 ChIP

Rev GGTGCCACCAATGTATGACC

-0.4 kb Fw TCCCAGATCAAAGTCATGTCA 64.5 ChIP

Rev GGCTCAACACCAGACTGAATC

-0.1 kb Fw AAAGTCAACCTTACTCCGCAAG 64.5 ChIP

Rev GCTGCCTGGACAGTATTCTCAC

+0.8 kb Fw TTGCCAAGTTTGTACAACTGCT 64.5 ChIP, RT-qPCR

Rev ACCTTGCAAAGACCATTCAGAT

**LANA region**

-0.5 kb Fw GTTTATAAGTCAGCCGGACCAA 64.5 ChIP

Rev GATATAACTCCGCCCTCCACTA

-0.3 kb Fw AAACAGAAACGGCCAATAACC 64.5 ChIP

Rev CCGTAAGGCACCCTTATCTTT

+0.6 kb Fw GAGTCTGGTGACGACTTGGAG 64.5 ChIP, RT-qPCR

Rev AGGAAGGCCAGACTCTTCAAC

**K2**

Pr Fw CATACGCAGCCAAGCTATCA 59 ChIP

Rev GCTAGCACAGCAAATTGAGA

in Fw TCACTGCGGGTTAATAGGATTT 64.5 ChIP, RT-qPCR

Rev CATGACGTCCACGTTTATCACT

**ORF56**

Pr Fw CGTTCTGAATAGACTGCAGT 59 ChIP

Rev CAAGATCACAGACTACCTGA

in Fw CACAGATTCCCGTCAATACAAA 64.5 ChIP, RT-qPCR

Rev GTATCTTCAGTAGGCGGCAGAG

**ORF8**

Pr Fw CATAGAGCCGAAGGACTGGA 59 ChIP

Rev ACCGATAATACCAGCTCTCT

in Fw CGACACGTACTCTTGTCTGACC 64.5 ChIP, RT-qPCR

Rev AAATAGAGTCCGCCTGTTGTGT

**ORF25**

Pr Fw AGTTGTCGGTGTCTATCTGT 59 ChIP

Rev TGCAGAGCGATACGCAGACT

in Fw ACAGTTTATGGCACGCATAGTG 64.5 ChIP, RT-qPCR

Rev GGTTCTCTGAATCTCGTCGTGT

**ORF64**

Pr Fw GCAGAAAGAACTTGAGCTCT 59 ChIP

Rev AGGCAGACCGGGAGTACGTT

in Fw CTTCCTCGAGGGCATCATATAC 64.5 ChIP, RT-qPCR

Rev TATACGGTGATGGACTTGATGG

**MYT1**

Fw GATTCTGTATTCATCATGGA 59 ChIP

Rev GCTGTGAGTGCTAGGATGTCT

Fw CACTGTGCGGAAGAGTTACT 64.5 RT-qPCR

Rev CTCATGCATGGCTAAGATCT

**HTF6**

Fw AAGCTGACCCAGGGAAGGAA 59 ChIP

Rev GATTGGATAAAGCTCCAGGA

**ACT**

Fw CCACAGCCAGAGGTCCTCAG 59 ChIP

Rev AGGAGCTCTTGGAGGGCATG

Fw TGGACATCCGCAAAGACCTG RT-qPCR

Rev CCGATCCACACGGAGTACTT

**p16** Fw GAAGGTCCCTCAGACATCCCC 64.5 RT-qPCR

Rev CCCTGTAGGACCTTCGGTGAC

**Zta** Fw GCTCCTGAGAATGCTTATCA 64.5 RT-qPCR

Rev AATTGTCTCCAGGTTGAGGT

**gp85** Fw ACTGCTATTCTGAGCACCAA 64.5 RT-qPCR

Rev TGGCAGTGATGTTCTGTGCA

**ORF45** Fw CCATACAGCGACCCTGATGA 58 RT-PCR

Rev CCGATTCTCTGACTCAATACT

**ORF48** Fw CCACATCTTCATAGAGCACAT 58 RT-PCR

Rev ATTGCATCACCAGGGTATCCA

**ORF50** Fw TTCGCCAAGCTCTACGTCCGA 58 RT-PCR

Rev TTGGACAGCTGTCGTTCAGAT

**K8** Fw GGTCTGTGAAACGGTCATTGA 58 RT-PCR

Rev TCTATGTAGTCGCCTCTTGGA

**shRNA target**

**EZH2** AAGACTCTGAATGCAGTTGCT
