# Supplementary material for: Epigenetic Analysis of KSHV Latent and Lytic Genomes
Source: PLoS Pathog. 2010 Jul 22;6(7):e1001013. doi: 10.1371/journal.ppat.1001013 (PMC2908616; doi:10.1371/journal.ppat.1001013)
Supplement: Table S2 — Genomic coordinates of the open reading frames of KSHV. IE = immediate early, E = early, L = late. Start codon coordinates indicate the first nucleotide of the KSHV genes and the stop codon coordinates indicate the last nucleotide in the stop codon of the KSHV genes. Coordinates are based on U75698.1 at GenBank. (0.05 MB DOC) [file ppat.1001013.s015.doc]

**Table S2.** Genomic coordinates of the KSHV genes

**Gene Start codon Stop codon Strand Class**

ORF K1 105 974 plus E

ORF 4 1142 2794 plus L

ORF 6 3210 6611 plus E

ORF 7 6628 8715 plus E

ORF 8 8699 11236 plus L

ORF 9 11363 14401 plus E

ORF 10 14519 15775 plus E

ORF 11 15790 17013 plus E

ORF K2 17875 17261 minus E

ORF 2 18553 17921 minus E

ORF K3 19609 18608 minus E

ORF 70 21104 20091 minus E

ORF K4 21832 21548 minus E

ORF K4.1 22529 22185 minus E

ORF K4.2 23147 22600 minus IE

ORF K5 26483 25713 minus E

ORF K6 27424 27137 minus E

ORF K7 28622 29002 plus E

ORF PAN RNA 28681 29742 plus E

ORF 16 30145 30672 plus E

ORF 17 32482 30821 minus E

ORF 18 32424 33197 plus L

ORF 19 34843 33194 minus E

ORF 20 35573 34611 minus L

ORF 21 35383 37125 plus E

ORF 22 37113 39305 plus L

ORF 23 40516 39302 minus L

ORF 24 42778 40520 minus L

ORF 25 42777 46907 plus L

ORF 26 46933 47850 plus L

ORF 27 47873 48745 plus L

ORF 28 48991 49299 plus L

ORF 29b 50417 49362 minus E

ORF 30 50623 50856 plus L

ORF 31 50763 51437 plus E

ORF 32 51404 52768 plus L

ORF 33 52761 53699 plus L

ORF 29a 54676 53738 minus E

ORF 34 54675 55658 plus L

ORF 35 55639 56091 plus L

ORF 36 55976 57310 plus E

ORF 37 57273 58733 plus E

ORF 38 58688 58873 plus L

ORF 39 60175 58973 minus L

ORF 40/41 60308 62444 plus E

ORF 42 63272 62436 minus L

ORF 43 64953 63136 minus L

ORF 44 64892 67258 plus E

ORF 45 68576 67353 minus IE

ORF 46 69404 68637 minus E

ORF 47 69915 69412 minus L

ORF 48 71381 70173 minus IE

ORF 49 72538 71630 minus E

ORF 50 71596 74629 plus IE

ORF K8 74850 75791 plus IE

ORF K8.1 75915 76695 plus L

ORF 52 77197 76802 minus L

ORF 53 77665 77333 minus L

ORF 54 77667 78623 plus E

ORF 55 79448 78765 minus L

ORF 56 79436 81967 plus E

ORF 57 82069 83544 plus E

ORF vIRF1 85209 83860 minus E

ORF vIRF4 88910 86074 minus E

ORF vIRF3 91394 89600 minus latent

ORF vIRF2 94127 91964 minus E

ORF 58 95544 94471 minus L

ORF 59 96739 95549 minus E

ORF 60 97787 96870 minus E

ORF 61 100194 97816 minus E

ORF 62 101194 100199 minus L

ORF 63 101208 103994 plus L

ORF 64 104000 111907 plus L

ORF 65 112443 111931 minus L

ORF 66 113759 112470 minus E

ORF 67 114508 113693 minus L

ORF 68 114768 116405 plus L

ORF 69 116669 117346 plus E

ORF K12 118101 117919 minus latent

ORF K13 122711 122145 minus latent

ORF 72 123567 122794 minus latent

ORF 73 127297 123809 minus latent

ORF K14 127884 128930 plus E

ORF 74 129372 130400 plus E

ORF 75 134441 130551 minus L

ORF K15 136772 134672 minus E

Notes: IE= immediate early, E= early, L= late. Start codon coordinates indicate the first nucleotide of the KSHV genes and the stop codon coordinates indicate the last nucleotide in the stop codon of the KSHV genes. Coordinates are based on U75698.1 at GenBank.
